# Inferring Viral Dynamics in Chronically HCV Infected Patients from the Spatial Distribution of Infected Hepatocytes

**DOI:** 10.1371/journal.pcbi.1003934

**Published:** 2014-11-13

**Authors:** Frederik Graw, Ashwin Balagopal, Abraham J. Kandathil, Stuart C. Ray, David L. Thomas, Ruy M. Ribeiro, Alan S. Perelson

**Affiliations:** 1 Los Alamos National Laboratory, Theoretical Biology and Biophysics, Los Alamos, New Mexico, United States of America; 2 Center for Modeling and Simulation in the Biosciences, Heidelberg University, Heidelberg, Germany; 3 Department of Medicine, Johns Hopkins University, Baltimore, Maryland, United States of America; University of Glasgow, United Kingdom

## Abstract

Chronic liver infection by hepatitis C virus (HCV) is a major public health concern. Despite partly successful treatment options, several aspects of intrahepatic HCV infection dynamics are still poorly understood, including the preferred mode of viral propagation, as well as the proportion of infected hepatocytes. Answers to these questions have important implications for the development of therapeutic interventions. In this study, we present methods to analyze the spatial distribution of infected hepatocytes obtained by single cell laser capture microdissection from liver biopsy samples of patients chronically infected with HCV. By characterizing the internal structure of clusters of infected cells, we are able to evaluate hypotheses about intrahepatic infection dynamics. We found that individual clusters on biopsy samples range in size from 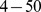 infected cells. In addition, the HCV RNA content in a cluster declines from the cell that presumably founded the cluster to cells at the maximal cluster extension. These observations support the idea that HCV infection in the liver is seeded randomly (e.g. from the blood) and then spreads locally. Assuming that the amount of intracellular HCV RNA is a proxy for how long a cell has been infected, we estimate based on models of intracellular HCV RNA replication and accumulation that cells in clusters have been infected on average for less than a week. Further, we do not find a relationship between the cluster size and the estimated cluster expansion time. Our method represents a novel approach to make inferences about infection dynamics in solid tissues from static spatial data.

## Author Summary

Around 170 million people worldwide are chronically infected with the hepatitis C virus (HCV). Although partly successful treatment options are available, several aspects of HCV infection dynamics within the liver are still poorly understood. How many hepatocytes are infected during chronic HCV infection? How does the virus propagate, and how do innate immune responses interfere with the spread of the virus? We developed mathematical and computational methods to study liver biopsy samples of patients chronically infected with HCV that were analyzed by single cell laser capture microdissection, to infer the spatial distribution of infected cells. With these methods, we find that infected cells on biopsy sections tend to occur in clusters comprising 4–50 hepatocytes, and, based on their amount of intracellular viral RNA, that these cells have been infected for less than a week. The observed HCV RNA profile within clusters of infected cells suggests that factors such as local immune responses could have shaped cluster expansion and intracellular viral replication. Our methods can be applied to various types of infections in order to infer infection dynamics from spatial data.

## Introduction

Around 170 million people worldwide are chronically infected with hepatitis C virus (HCV), representing a major public health problem [1]. Chronic HCV infection can lead to liver cirrhosis, hepatocellular carcinoma and liver failure, and it represents the leading cause for liver transplantation in Western countries [2]. Despite successful treatment options using mostly type I interferon-*α* (IFN-*α*) or newer direct-acting antiviral agents, several aspects of HCV infection dynamics are still unknown. For example, does the virus propagate preferentially by cell-to-cell transmission or via diffusion of viral particles? Do innate immune responses interfere with the spatial spread of the infection? And what fraction of hepatocytes are infected during the acute and chronic stages of infection?

To answer these questions, a better understanding of the *in vivo* infection process is needed. As appropriate animal models for HCV infection are lacking, inferring *in vivo* infection dynamics from clinical data has relied on mathematical models that describe the interaction of hepatocytes with viral particles [3]–[8]. Mathematical modeling of viral load dynamics in combination with data on treatment with IFN-*α* and direct-acting antivirals has helped to reveal and quantify aspects of the infection process, such as the half-life of viral particles and the loss rate of infected hepatocytes under treatment [3], [6], [9], [10]. In addition, models have quantified the necessary treatment efficacy to clear the virus [5], [11].

Existing models have been fit to HCV RNA levels measured in the serum of patients. Measurements of viral levels in the liver and in particular of HCV RNA levels within cells of the liver have generally been lacking. Advances in techniques, such as two-photon microscopy [12], [13] and laser capture microdissection [14], now allow one to visualize and analyze HCV infection in the liver at the cellular level. Using single cell laser capture microdissection (scLCM), it is possible to determine the HCV RNA content in single hepatocytes from liver biopsies of HCV infected patients as well as the spatial relationships among infected cells [15]. Analyzing regular grids of 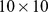 hepatocytes we found that infected hepatocytes tend to occur in clusters [15], in agreement with other studies reporting a focal distribution of HCV RNA in infected liver tissue [12], [14], [16], [17]. However, patients differ in their individual viral load, as well as in the frequency of hepatocytes infected.

To extend our previous observation of a spatial heterogeneous distribution of infected hepatocytes [15], we now develop statistical methods to characterize properties of clusters of infected hepatocytes in more detail, e.g. in terms of cluster size and intracellular HCV RNA levels. Analyzing data from 4 chronically infected patients [15], we find that clusters of infected cells comprise between 4 and 50 cells in the plane of the liver biopsies. These sizes are comparable to the range of cluster sizes observed in *in vitro* experiments on Huh-7.5 cells under conditions only allowing cell-to-cell transmission [18]. In addition, we find that the level of intracellular viral RNA declines in infected cells at increasing distance from the cell that presumably founded the cluster [12], [19]. Using intracellular HCV RNA content as a proxy for the time since infection in a given cell, this suggests that cells closer to the founder cell of the cluster have been infected for a longer time than those in the periphery. Both of these observations suggest that viral infection once seeded spreads locally, supporting cell-to-cell transmission [12], [20] or viral release from an infected cell with rapid binding to and infection of neighboring cells. We then used mathematical models to describe intracellular viral replication and accumulation of viral RNA. Applying these models to interpret the data, we do not find a relationship between the observed cluster size and the estimated time that the cluster has been expanding, suggesting that individual cellular factors might influence cluster growth. We also estimate that the cells in the detected clusters have been infected on average for less than a week. This finding is consistent with previous estimates of the mean lifetime of HCV infected cells [21], [22]. Overall, our study presents a set of novel methods to infer *in vivo* viral dynamics of chronic HCV infection in the human liver based on liver biopsy samples.

## Results

### Determining clusters of infected cells

In a previous analysis of two-dimensional 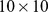 grids of hepatocytes analyzed by scLCM, we obtained evidence for clustering of HCV infected cells in the liver [15]. Determining the size of individual clusters visually based on the actual grid data is difficult as we are only analyzing a small fraction of tissue. Infected cells at the edge of the sampling area might be part of a larger cluster that extends outside the sampling region. In this study, we apply enhanced methods of spatial statistics to the data in order to (1) estimate the size of clusters accounting for edge effects due to the limited sampling area, and (2) characterize the structure of clusters of infected cells in more detail.

**Figure 1:**
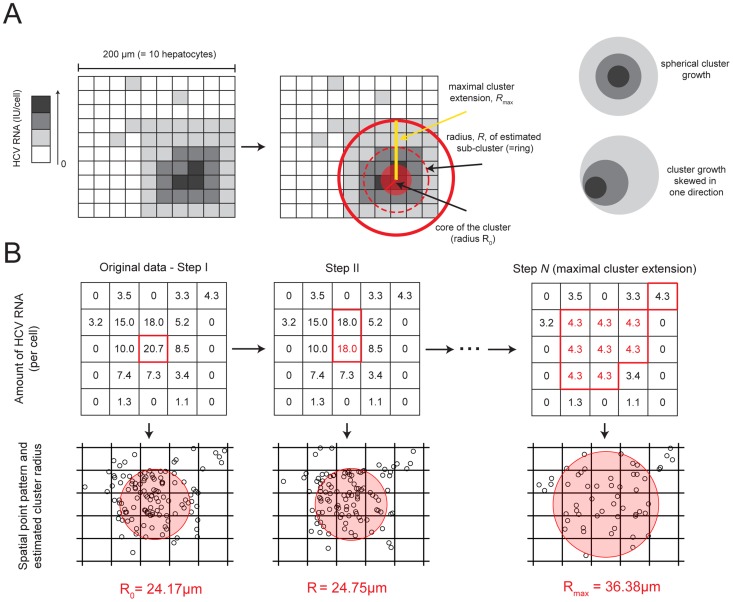
Sketch of the characterization of clusters of infected cells. (**A**) Example of measured data in a 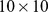 grid of cells with the HCV RNA content per cell (left) and sketch of the ring structure of the cluster (right) as it would be defined by the algorithm shown in (**B**). Darker shading of cells indicates a higher amount of HCV RNA. The fitting procedure of the Matérn cluster process to estimate the domain radius 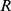 accounts for edge effects due to sampling, i.e., only parts of the cluster might be visible on the grid of liver tissue analyzed by scLCM. The sketch in (**A**) shows an example for a cluster that grew spherically. The algorithm also allows for cluster growth that is skewed in one direction. (**B**) Example of the algorithm to determine the “ring structure” of a cluster of infected cells for a 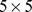 grid of cells. The measurements of HCV RNA per cell are transformed into a spatial point pattern (see *Materials & Methods*). The amount of HCV RNA in those cells with the maximal HCV RNA content is subsequently reduced to the next lower level (red color) (Step I–Step II). For each of the different steps, 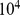 spatial point patterns are produced, and a *Matérn cluster process* is fitted to each pattern to estimate 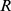. Step 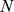 shows the last step before the cutoff criterion for the maximal cluster extension. For all subsequent steps of this example Pearson's chi-squared statistic for the point patterns indicated spatial heterogeneity for less than 95% of the 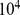 bootstrap samples.

If hepatocytes in the liver were infected completely at random, for example due to rapid seeding from the blood, we would expect homogeneous infection and no clusters. Since we observe clusters of infection [15], we make the next most parsimonious assumption that viral spread *in vivo* is a combination of random spatially scattered infection of some cells that seed the cluster (possibly from virus in the blood) followed by predominantly random local spread from these cells. We assume that seeding of the cluster centers follows a Poisson process, with the mean number of clusters per unit area equal to 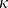, and the number of cells in each cluster also following a Poisson process, with the mean number of cells in each cluster equal to 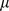. This compound Poisson spatial distribution is called a Matérn cluster process [23], [24]. A Matérn cluster process assumes that the units of a cluster are distributed within a radial disc with domain radius 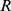 (Figure 1A). Assuming this regular cluster structure allows us to account for edge effects due to the small sampling area, i.e., that only parts of a cluster were sampled on the grid [25].

To determine individual cluster sizes, we fit a Matérn cluster process to each of the two-dimensional 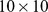 grids of hepatocytes analyzed by scLCM to estimate the domain radius 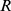. We proceed by first randomly distributing the HCV RNA content of each cell over the space occupied by the cell in the grid and then fitting a Matérn cluster process to these spatial point patterns (Figure 1B and Text S1), rather than fitting to the spatial distribution of infected hepatocytes. This is done as (i) a Matérn cluster process assumes continuous space, and (ii) because we are also interested in the internal structure of the cluster (*see below*). We note that the random distribution of HCV molecules within a cell is an artificial construct to allow us to estimate the radius of the cluster, when in fact HCV replication likely occurs in localized structures in the cell (reviewed in [26]). To make sure that the particular distribution of HCV RNA inside the infected cell does not affect the results, we repeat the procedure of random allocation of HCV RNA inside the cell 10,000 times, thus obtaining that many bootstrap replicates of our data set.

With this transformation, the algorithm detects regions with similar densities of HCV RNA. It is possible that cells with low amounts of HCV RNA that are adjacent to cells with higher levels of HCV RNA would not be counted as part of a cluster due to the difference in the density of viral RNA. To account for this potential artifact of the algorithm, we estimated the domain radius 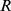 of a cluster based on the minimum level of HCV RNA in all cells assumed to belong to the cluster (Figure 1B and *Materials & Methods* for details). Since we do not know *a priori* which cells belong to the cluster, our estimate is done iteratively. We start with the cell with the highest HCV RNA content and estimate the cluster radius 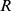 for the obtained point pattern (Figure 1B). In succession, we assume that the cell with the next highest content would be the cell with the minimal level of HCV RNA in the cluster, we reduce higher levels of HCV RNA to this level (Figure 1B) and again fit a Matérn Cluster process to the new point pattern. We stop when inclusion of the next cell would result in an apparent homogeneous distribution of infected cells as determined by the Quadrant-Count method (see *Supporting Information*, Text S1). At this point, we are not detecting individual clusters, because we included so many cells that there is only one large homogeneous cluster. Detection of the maximal cluster size, and thus the average number of clusters per sampled grid, is independent of the assumed founder cell as we are identifying regions of cells with similar densities of HCV RNA. Our algorithm allows all infected cells to be counted with equivalent weights, and because we estimate the cluster radius in iterative steps, we characterize the structure of each cluster analogous to the ring-structure of a tree trunk, where successive rings have similar spatial density of HCV RNA (Figure 1). Note that we perform this iterative procedure with all 10,000 bootstrap replicates.

**Figure 2:**
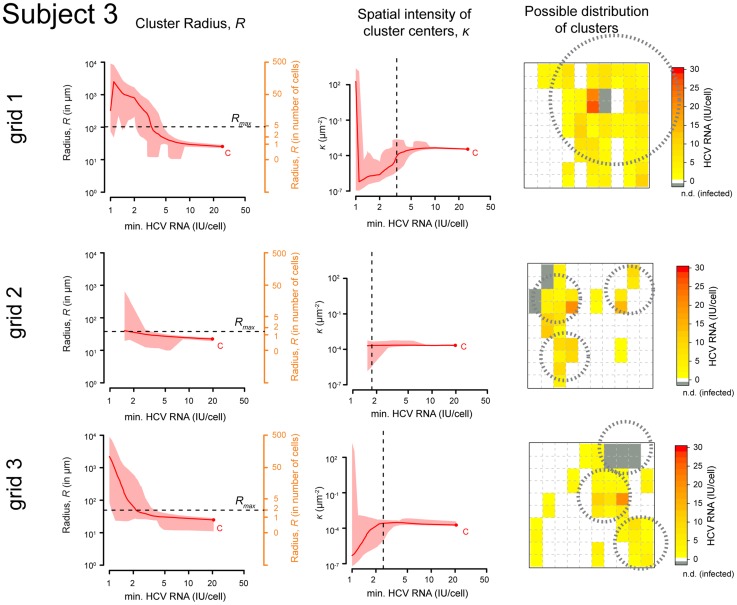
Estimates of domain radius and spatial intensity. Estimates of the domain radius 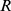 (left panel) and the spatial intensity 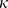 (middle panel) dependent on the minimal HCV RNA content of cells in the clusters for each of the three different grids on the sections of subject 3. Plots should be read from the right to the left as the algorithm starts at point 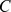, the maximal amount of HCV RNA measured in an infected cell on the indicated grid. The domain radius of the cluster, 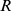, is given on a continuous scale, as well as an estimate of the radius in number of cells. The red line is the median over 10,000 bootstrap replicates of fitting a *Matérn cluster process* to the data as described in *Materials & Methods*. The red area denotes the 95%-quantiles of the estimates. The dashed horizontal (left panels) and vertical (middle panels) lines indicate the cutoff of the algorithm, i.e., the maximal extension of the cluster, 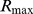. The right panels show the observed spatial distribution of infected hepatocytes together with the measured HCV RNA amount. Grey squares indicate infected cells for which the actual HCV RNA amount could not be determined (n.d.). For the analysis, the HCV RNA amount of these cells was extrapolated according to different methods (see *Materials & Methods*). Plots are combined with a possible distribution of the clusters showing for each the maximal cluster radius, 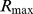 (dashed circles). On grid 2 and 3, the estimated spatial intensity 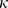 is about 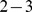 times higher than on grid 1, predicting the existence of several individual clusters on these sections.

We applied the above procedure to each grid of each subject individually. In Figure 2, we show, for subject 3, the estimates of the cluster radius, 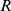, and the corresponding expected number of cluster centers per unit area, 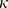, as a function of the minimal HCV RNA content of cells in the clusters at each iteration of our algorithm (from right-to-left). The maximal cluster extension, 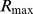, is found when the mean number of cluster centers, 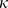, starts to decrease from its more or less constant value (Figure 2, reading right-to-left). A decreasing 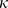 indicates fewer and larger clusters, a more homogeneous distribution, and in the limit indicates that all cells on the grid belong to one single cluster. Since we previously determined that infected cells are clustered [15], such inference of a homogeneous distribution is unreasonable. For each iteration we analyze, in each of our bootstrap replicates, if the resulting spatial distribution still indicates clustering or not. The iterative process is stopped when we start finding a homogeneous spatial distribution, that is when less than 95% of the replicates show evidence of clustering. On grid 1 of subject 3, the algorithm detects one large cluster, while on the other two grids, the existence of several smaller clusters of infected cells is predicted, with the estimated mean number of clusters, 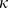, on these grids being 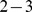 times larger than on grid 1. The corresponding figures for all other subjects are shown in the *Supporting Information* (Figures S1 and S2–S4).

### Cluster characteristics

The algorithm provides estimates of the cluster radius and the number of clusters in each grid, however it does not explicitly show where each cluster is located. Based on the results for 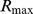 and 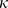 for subject 3, in the right panel of Figure 2, we infer a possible spatial distribution of clusters of infected cells on each 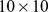 grid for this subject as shown. Overall, with this method we find that our 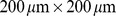 sections of liver biopsies contain between ∼1 and ∼45 clusters of infected cells, which have radii between 12.9 *µ*m and 103 *µ*m (Table 1).

**Table 1:** Characteristics of determined clusters.

subject	grid	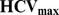 (in IU/cell)	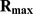 (in 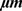 )		Cluster Size (in cells)		nbr of clusters	total HCV RNA (in IU/cell)	
1	1	22.8	12.9	(5.2,22.1)	1.5	(1,2)	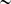 45	34.2	(22.8,45.6)
	2	5.5	36.7	(20.3,407.1)	12.5	(11,14)	2–3	39.5	(33.3,46.3)
	3	7.7	67.3	(23.6,642.3)	41	(36,46)	1	129.7	(115.5,144.1)
2	1	50.3	39.5	(25.1,105.7)	14	(12,16)	1–2	139.5	(110.3,197.2)
	2	5.0	23.9	(16.7,101.9)	5	(4,6)	3	25.1	(20.0,30.1)
	3	8.8	53.1	(19.6,747.2)	26	(22,29)	1	71.9	(47.1,99.0)
3	1	25.9	103.0	(12.0,514.0)	95	(83,107)	1	476.4	(431.3,523.2)
	2	19.7	37.9	(20.9,389.5)	13	(11,15)	2–3	85.6	(62.5,113.9)
	3	20.7	41.5	(26.0,266.8)	16	(14,18)	3	129.0	(99.1,160.5)
4	1	19.8	21.8	(15.2,37.2)	4.5	(4,5)	5–6	89.0	(79.0,98.8)
	2	20.0	27.7	(18.0,758.5)	7	(6,8)	1–2	139.8	(119.8,159.8)

For each of the different subjects and grids, we give the characteristics of the determined clusters in terms of maximal HCV RNA content in a cell belonging to this cluster, i.e., the founder cell, HCV_max_ and the maximal cluster extension, 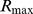 (median of 10,000 bootstrap replicates (Fig. 2)). In addition, the estimated number of clusters per grid and, based on 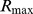, the average cluster size and the average total HCV RNA content in clusters on this grid are given. Numbers in brackets represent the 2.5% and 97.5% percentiles based on 10,000 bootstrap replicates (see *Materials & Methods* for a detailed description of the calculation of these values). For each grid, the clusters including the cell with the highest amount of intracellular HCV RNA were considered.

To determine the size of a cluster in terms of numbers of cells, we transform the cluster radius 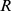 back into an actual number of hepatocytes, 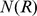. The radius 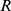 was estimated searching for areas with similar densities of HCV RNA and assuming a circular structure of the cluster, but hepatocytes themselves are better described by a square geometry (Figure 1B). Thus for a given radius we estimate the number of cells inside a circle of radius 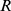 and inside a square with sides of length 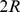 (where 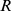 was estimated based on our algorithm above). Note that by definition most hepatocytes in a cluster are infected, however some uninfected cells could also be counted in a cluster. This is because clusters are defined as a circle around the seeding infected cell, and uninfected cells could be in close proximity to infected cells and by chance were not (yet) infected (see Figure 1B, last step). The minimum number of cells belonging to a cluster is determined by 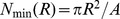, where the area of the cluster is given by a disc with radius 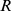, and 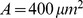 denotes the area of a hepatocyte. The maximum number of cells in this cluster is estimated by assuming the area of the cluster is given by a square with edge length 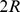, hence, 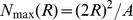. For example, the estimated number of hepatocytes in a cluster with a maximal cluster extension of 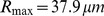 as determined on grid 2 of subject 3 (Figure 2) ranges between 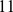 and 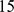 cells.

We found that clusters comprise between 4–50 infected hepatocytes, not considering the two extreme cases (grid 1 of subject 1 and grid 1 of subject 3) that have to be taken with care (Table 1). For the maximal cluster size of 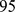 cells estimated for grid 1 of subject 3 (Table 1), the estimate of 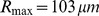 is close to the reliability threshold for 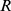 (see *Materials & Methods*), since a substantial part of the cluster is outside the sampled region. For grid 1 of subject 1, we do not find evidence for a spatially heterogeneous distribution of infected cells (see also Figure 5B of [15]). Therefore, the cluster detection algorithm is affected and determines a “cluster” for each infected cell (see Figure S1 and Figure S2). An important point is that we are only analyzing 2D sections, and most likely clusters are defined in 3D. Thus, the total number of cells in a cluster will be larger than our minimal estimates.

### Cluster size and HCV RNA profile

**Figure 3:**
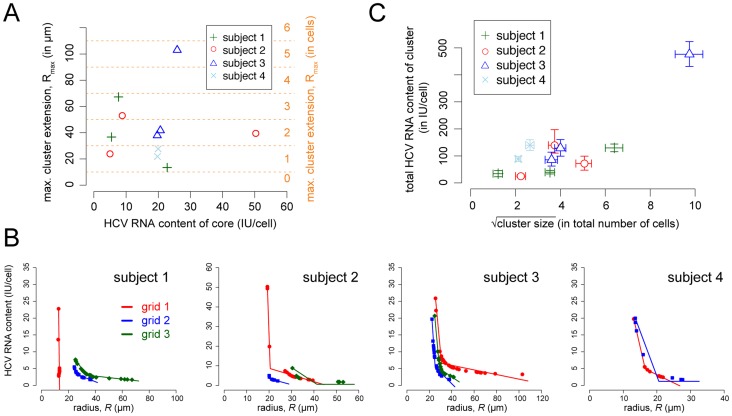
Relationship between cluster extension and HCV RNA content. (**A**) The maximal extension of the clusters, 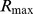, characterized on the different grids in relation to the maximal cellular HCV RNA content in the cells belonging to this cluster. For each grid, only the clusters including the cell with the highest amount of intracellular HCV RNA were considered. Different patients are indicated by different symbols and colors. The radius 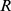 is given on a continuous scale, as well as converted into number of cells assuming radial spread. (**B**) The level of the HCV RNA content in hepatocytes with increasing cluster extension as characterized by Fig. 2. Dots correspond to the radii estimated during the iterative process of cluster determination. The individual results for all patients and all different sections per patient (grid 1 *red*, grid 2 *blue*, grid 3 *green*) are shown. Lines indicate the best fit of a model assuming a biphasic linear decrease of the intracellular HCV RNA content with increasing cluster extension (see Table 4). (**C**) The total HCV RNA content in an inferred cluster, i.e., the sum of the HCV RNA content in all hepatocytes belonging to a cluster versus the square root of the cluster size measured in number of cells. Each point is the result of 10,000 simulated clusters based on the characteristics for each of the different clusters per patient per grid as described in *Materials & Methods*. Symbols indicate the mean of total HCV RNA content and cluster size, arrows determine the 2.5% and 97.5% percentiles of the 10,000 bootstrap replicates. Corresponding values are given in Table 1.

After defining the clusters and their sizes, we analyzed the profile of the intracellular HCV RNA level within the clusters, i.e., the viral landscape or viroscape [15]. The relationship between the maximal radius of a cluster of infected cells and the HCV RNA content in the cell that presumably founded the cluster is shown in detail in Figure 3A. For example, the hepatocyte with the highest amount of HCV RNA on grid 3 of subject 2 contained 8.8 IU/cell, and the corresponding cluster of infected cells reached a maximal radius of 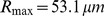 (see Table 1). The mean cluster radius among all patients was 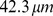 (95%-CI [26.7, 57.9]) with variability between and within patients. There was no significant relationship between the maximal cluster radius and the HCV RNA content in the assumed founder cell of the cluster, i.e., the cell with the highest HCV RNA content (Figure 3A 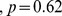, linear mixed effects model). This result does not change if grids 1 of subject 1 and 3, which could be outliers (see above), are neglected.

The iterative algorithm that was used to determine the maximal cluster radius (Figure 1) also describes the HCV RNA content in a cell as a function of the distance to the core of the cluster assuming radial spread. We find that the viroscape of a cluster of infected cells, i.e., the topography of the amount of intracellular viral RNA, can be characterized by a biphasic decline in the amount of HCV RNA content of cells with increasing cluster extension (Figure 3B).

As no relation could be found between the maximal cluster extension and the HCV RNA content in the assumed founder cell of the cluster (Figure 3A), we then asked if there was a relationship between the total amount of HCV RNA in a cluster and the actual size of a cluster (i.e., number of cells in the cluster). To this end, we have to convert continuous characteristics like the radii of the cluster, 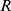, back into discrete number of cells. For consistency with the method above, we constructed 10,000 random bootstrap replicates of the observed clusters. To maintain the properties in the data, these replicates were defined consistent with individual cluster characteristics, namely their maximal cluster extension in *µ*m (Table 1) and the observed biphasic decline in HCV RNA with increasing cluster extension (Figure 3B) (see *Materials & Methods*). The results of these analyses do not indicate a significant correlation between cluster size (in number of cells) and the total amount of HCV RNA in a cluster (Figure 3C, Linear-mixed effects model, 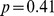, neglecting grids 1 of subject 1 and 3). Previously detected correlations between cluster size and viral load (see Fig. 6 of [15]) are largely affected by grid 1 of subject 3.

### HCV RNA profile and infection dynamics

**Figure 4:**
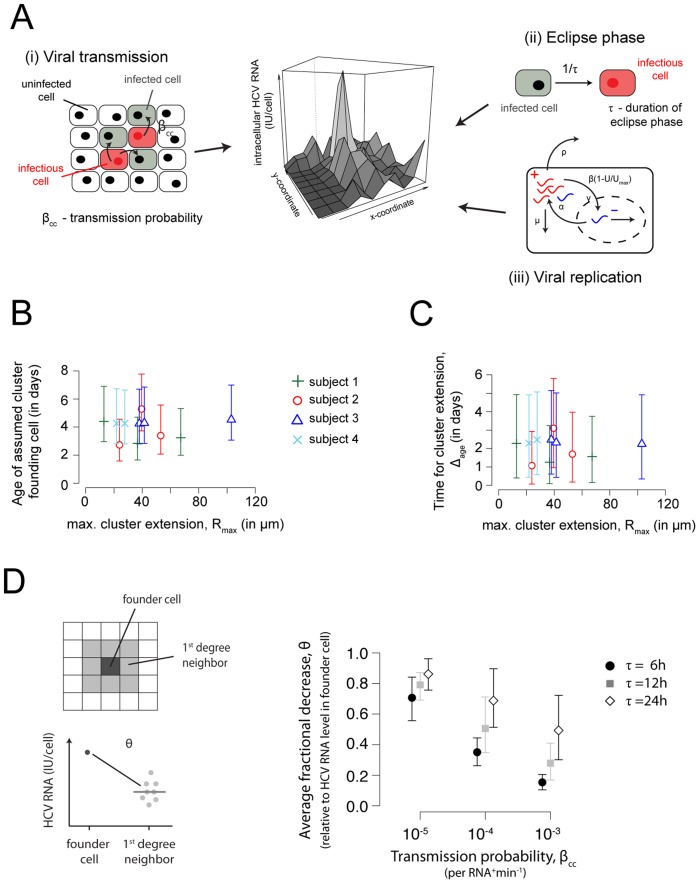
Cluster structure and age of infection. (**A**) Factors influencing the observed viral landscape in a cluster including (i) viral transmission, (ii) the viral eclipse phase, and (iii) intracellular viral replication. (**B, C**) Relationship between the maximal cluster extension and the age of the assumed founder cell of the cluster (**B**) and the time until this cluster extension was possibly reached, 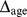 (**C**), i.e., the difference between the age of the assumed founder cell and cells that were more recently added to the cluster. Estimates were obtained using the model on intracellular HCV replication discussed in the text. The mean and the 2.5% and 97.5% percentiles of 10,000 bootstrap replicates for each individual cluster are shown. (**D**) Comparison of the average fractional decrease in intracellular HCV RNA, 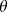, between the founder cell and the surrounding cells of a cluster comprising 9 cells. Expansion of individual foci was simulated in a 2D-grid with different assumptions for the average duration of the eclipse phase 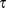, and the probability for viral transmission, 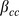. We simulated 1000 individual foci and selected all foci showing clusters comprising 9 cells with radial spread. The average fractional decrease is calculated relative to the amount of viral RNA within the founder cell of the cluster. The mean and 95%-percentiles are shown. For a detailed description of the simulation environment see *Supporting Information* (Text S3).

Several aspects of viral replication and transmission dynamics should influence the observed HCV RNA profile within a cluster of infected cells. These include the rate at which neighboring cells get infected, the average time it takes for a cell to start viral replication, the rate at which the virus replicates, the rate at which intracellular HCV RNA is degraded, and the lifespan of infected cells (Figure 4A).

We assume that the amount of HCV RNA inside an infected hepatocyte can be used as a surrogate for the time a cell has been infected, the so called age of infection, 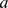. That is, hepatocytes with higher amounts of HCV RNA most likely have been infected for longer. After infection, a number of events have to occur, taking time 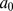, before viral RNA replication starts. Following our previous work [27], we assume that after 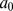, each viral RNA can be copied in ∼6*h*, which would be the doubling time of the amount of viral RNA inside a cell, if there was no export of virions or degradation of viral RNA. However, we have estimated that on average 75% of new viral RNA is exported in virions [27], although this fraction could vary over time. In our data, we find hepatocytes with up to ∼50 IU/cell, corresponding to ∼100 HCV RNA copies/cell (1 IU
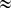
1.96 genome copies [15]). This value is consistent with other *ex vivo* studies [14], [17], [19]. With these parameters, we simulate the stochastic replication dynamics of HCV within a cell [27] following the accumulation of HCV RNA within the cell. Varying 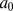 between 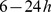, we estimate that cells reach a level of 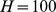 HCV RNA copies/cell on average 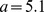 days p.i. (95%-CI [3.5 days, 7.7 days]).

Based on the observed HCV RNA content, we estimate that cells in the liver samples are infected for less than a week (Figure 4B and Table 2, see also Text S2). The cells assumed to build the core of the individual clusters, i.e., earliest infected and containing the largest amount of intracellular viral RNA, are estimated to be infected for on average ∼4 days (Table 2). Calculating the elapsed time between infection of the cells with the largest amount and the lowest amount of intracellular viral RNA within a cluster, 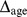, i.e., the time the cluster has been expanding, we estimate that the observed clusters of infected cells were formed on average in 2 days (Table 2 and Figure 4C). We do not find a relationship between the maximal cluster size and the estimated age of infection of the hepatocyte that presumably founded the cluster, nor between the maximal cluster extension and the time a cluster has been expanding, 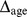 (Figure 4B,C, 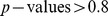, linear mixed-effects model). This could indicate variability in viral propagation compared between and within patients, possibly due to individual and locally available host factors as well as individual viral characteristics, such as the ability to spread cell-to-cell and the speed of spread.

**Table 2:** HCV RNA and age of infection.

subject	grid	Age founder cell, 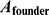 (in days)		Age difference, 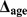 (in days)	
1	1	4.4	(2.9, 6.9)	2.3	(0.4, 5.0)
	2	2.8	(1.6, 4.7)	1.3	(0.1, 4.9)
	3	3.2	(1.9, 5.3)	1.6	(0.2, 3.2)
2	1	5.3	(3.7, 7.8)	3.1	(0.9, 5.8)
	2	2.7	(1.5, 4.6)	1.0	(0.0, 2.9)
	3	3.4	(2.0, 5.6)	1.7	(0.1, 4.0)
3	1	4.5	(3.0, 7.0)	2.2	(0.3, 4.9)
	2	4.3	(2.8, 6.7)	2.5	(0.6, 5.1)
	3	4.3	(2.8, 6.9)	2.3	(0.4, 5.0)
4	1	4.3	(2.8, 6.7)	2.3	(0.4, 4.9)
	2	4.3	(2.8, 6.3)	2.5	(0.5, 5.1)

For each of the different subjects and grids, we estimated the age of infection of cells based on their HCV RNA content using the stochastic model of HCV viral replication as described in [27] with 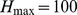 copies per cell and varying 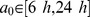. The mean, and the 2.5% and 97.5% percentiles based on 10,000 bootstrap replicates are shown. The age of the assumed founder cell, 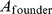, and the difference in age between the founding cell and those that were more recently added to the cluster in the periphery, 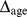, are shown. For each grid, the cluster including the cell with the highest amount of intracellular HCV RNA was considered.

The ratio between the rate at which neighboring cells get infected and the rate at which HCV RNA accumulates within an infected cell influences the steepness of the viroscape with increasing cluster size. For example, if the virus has a high local transmission probability combined with a short time between cell infection and new virion production, the so-called eclipse phase, we would expect a more uniform distribution of HCV RNA content in individual cells, i.e. a flatter viroscape. In contrast, a long eclipse phase combined with a low transmission probability will lead to increased accumulation of HCV RNA within the cells that got infected early on, showing larger differences in the HCV RNA content between the cells within a cluster. We simulated cluster expansion by local spread within a two-dimensional lattice of hepatocytes to investigate how the transmission probability per intracellular viral RNA per minute and the duration of the eclipse phase would influence the viral profiles in a cluster (Text S3). Calculating the average fractional decrease between the viral RNA within the founder cell and the HCV RNA amount in the surrounding cells in a cluster of size 9, we observe that the largest declines are obtained for long eclipse phases, 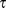, and low cell-to-cell transmission probabilities per positive strand intracellular HCV RNA per minute, 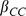 (Figure 4D). The viral profiles in our data show a biphasic decay (Figure 3B), with an average first-phase fractional decrease of intracellular HCV RNA among all patients (Figure 4D) of 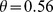 (range [0.30,0.91]) relative to the maximal amount of HCV RNA in the founder cell of the cluster. This means that the HCV RNA level in direct neighbors of the core of the cluster is on average ∼40%, 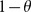, of the HCV RNA level within the core, i.e., that the intracellular HCV RNA level decreases by ∼60%. We can recover these observations in our simulations if the probability for virus transmission per intracellular viral RNA per minute, 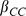, lies approximately between 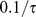 and 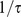, where 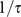 defines the rate at which infected cells become infectious, i.e., start producing virus (Figure 4D).

### Inferring infection in the whole liver

For each patient, two to three 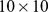 grids of hepatocytes were analyzed by scLCM. Thus, only a small number of hepatocytes (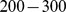 cells) was sampled and analyzed in comparison to the estimated total number of 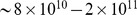 hepatocytes in a human liver [28]–[31]. To determine if the infection patterns in the sampled sections of liver tissue are consistent with the infection dynamics in the entire liver, we compared the measured serum viral load for each patient to the expected serum viral load from the observed liver sections using an age-structured model of HCV infection (*Materials and Methods* and [9]): With an export rate, 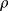, of intracellular viral RNA as virions and a clearance rate of extracellular virus, 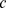, the viral load per ml of serum produced by 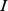 infected hepatocytes can be estimated by 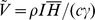, where 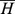 denotes the average amount of intracellular HCV RNA per infected hepatocyte and 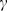 is a scaling constant, relating the total amount of virus in the body, 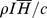, to virus per ml. We used our data to determine 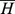 and 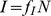, with 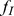 denoting the observed fraction of infected hepatocytes and 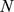 the total number of hepatocytes in a human liver. With the previously estimated values of 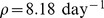 and 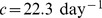 [10], and with 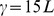, the average total extracellular fluid volume for an individual, we estimate 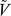 based on the sampled liver biopsies (Table 3).

**Table 3:** Subject characteristics.

subject	Age (yrs)	sex	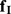 ( 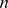 )	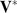 (log 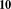 IU/ml)	metavir	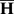 (IU/cell)	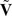 (log 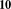 IU/ml)
1	43	female	0.40 (258)	7.40	0	3.03 (2.51,3.55)	6.77 (6.68,6.84)
2	47	male	0.20 (254)	6.87	0	5.14 (2.45,7.83)	6.69 (6.37,6.88)
3	44	male	0.43 (288)	7.02	1	4.80 (4.06,5.54)	7.00 (6.93,7.07)
4	40	male	0.20 (200)	6.90	1	3.76 (2.10,5.41)	6.57 (6.31,6.73)

For each subject, we give the frequency of infected hepatocytes, 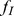, the total number of hepatocytes analyzed, 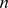, the measured viral load, 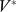, and their disease stage (metavir). For 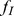 and 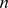, we considered only cells where the HCV RNA amount could be determined. 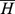 denotes the average HCV RNA content per infected cell, with the 95% confidence intervals calculated by mean 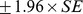. The 95% confidence intervals for the estimated viral load 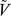 assuming 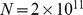 hepatocytes in total are given by the confidence limits of 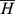.

**Table 4:** Decline of viroscape profile.

subject	grid	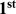 phase decay, 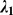 (in IU 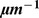 )		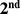 phase decay, 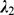 (in 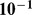 IU 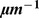 )		phase change, 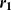 (in 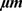 )		slope, 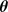 (in % of 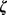 )
1	1	32.49	-	9.24	-	40.5	-	(*)
	2	1.15	(1.04,1.25)	1.59	(1.35,1.83)	26.3	(26.0,26.6)	30
	3	0.57	(0.55,0.58)	0.45	(0.39,0.51)	33.3	(33.0,33.6)	35
2	1	33.21	(32.83,33.60)	3.41	(3.15,3.66)	20.5	(20.5,20.6)	81
	2	11.39	(3.73,19.04)	3.14	(0.56,5.70)	20.2	(20.0,20.3)	31
	3	0.76	-	0.0	-	41.4	-	45
3	1	3.58	(3.55,3.61)	0.68	(0.65,0.71)	30.9	(30.8,31.0)	48
	2	5.03	(4.98,5.08)	3.35	(3.15,3.55)	25.2	(25.1,25.3)	57
	3	3.64	(3.57,3.70)	2.26	(2.05,2.47)	29.1	(29.0,29.2)	52
4	1	4.36	(4.26,4.46)	4.86	(3.87,5.84)	16.5	(16.4,16.7)	91
	2	2.68	-	0.0	-	20.6	-	91

Estimates for the model assuming a biphasic linear decline in the intracellular amount of HCV RNA within a cluster with increasing cluster extension (Eq. (1)). The average fractional decrease 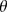 of the first phase decline relative to the maximal amount of HCV RNA 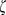 in core of radius 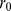 is then calculated by 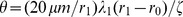. The first slide of subject 1 (*) is not considered due to the observed irregular viroscape profile impairing a reliable fit to the data. The 95% confidence intervals for the estimates are calculated based on the standard error approximated by the Hessian matrix.

For each subject, estimates for 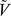 are in a range consistent with, but slightly lower than, the measured serum viral load (Table 3). On average our estimates are 0.3 log (or 5%) lower than the measured viral load. A possible explanation for this lower estimate is a higher concentration of HCV RNA in plasma than in other body fluids [32], as we assumed a homogeneous distribution throughout the extracellular fluid when calculating 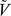. The other possibility is that the value of 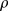 could vary among patients, whereas we are using an average estimate for all of them. We note that although 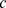 could also vary, recent estimates show that *c*∼22 day^−1^ with little inter-patient variability [10]. In summary, based on this analysis it seems reasonable to assume that the patterns of infection (fraction of infected cells, density and level of intracellular HCV RNA) in the liver sections are an appropriate and consistent representation of the infection in the whole liver.

## Discussion

In previous work, we showed that infected hepatocytes tend to occur in clusters [15]. However, our previous cluster detection methods [15] were not able to specify cluster characteristics, such as the cluster size and the dynamics of cluster formation. Here we developed statistical methods to determine the properties of such clusters of infected cells identified by scLCM. With our approach, which is based on assuming infected cells are distributed according to a Matérn cluster process, we are able to systematically determine the size and the internal structure of clusters. In comparison to other clustering methods [33], [34], our stepwise procedure allows us to address the dynamics of the cluster building process based on the varying but interdependent levels of intracellular viral RNA.

Applying our method to liver biopsy samples from four different chronically infected HCV patients analyzed by scLCM, we find that clusters of infected hepatocytes in a biopsy section comprise between 4 and 50 cells (Table 1). While we show results based on a Matérn cluster process that assumes radial cluster shapes, we also fitted a Thomas process, which allows for normally distributed “offspring” around cluster centers, to the data [23]. This did not change our results in terms of cluster size or cluster frequency (*not shown*). One caveat is that we are studying 2D sections, while *in vivo* the clusters of infection would be 3D. It is not easy to expand our results to that situation, because each 2D section could represent a different cross-section of the corresponding 3D cluster. Sequencing the intracellular HCV RNA in future studies could indicate whether viruses from nearby clusters are distinct, and therefore originate from separate clusters, or whether they represent cross-sections of irregular 3D clusters.

It is interesting to compare our results to 2D infection *in vitro*. In an experiment using HCV infected Huh-7.5 cell lines, Lacek et al. [18] found cluster sizes ranging between 1–40 cells around 2 days after the initial infection, similar to the sizes and timing we report. However, when blocking local transmission by appropriate monoclonal antibodies, they found a significant reduction in cluster sizes [18]. At the highest antibody concentration used, the maximum cluster size was reduced to 5 cells (see Fig. 3A in [18]). The agreement of the cluster sizes found *in vitro* with the cluster sizes found in our study, as well as the findings of others that HCV infected cells tend to occur in clusters [12], [14], [15], [17] support the hypothesis that local spread of HCV is an important contributor to the patterns we observed.

The hypothesis of local spread is also corroborated by our second result, indicating that the HCV RNA amount within infected hepatocytes of a cluster declines with increasing cluster extension (Fig. 3B). Using the HCV RNA amount inside an infected hepatocyte as a surrogate for the time since infection, this observation supports the notion that HCV spreads locally to neighboring cells and then begins replicating within those cells. However, we cannot distinguish the hypothesis that the local spread is mediated by diffusing viral particles that rapidly bind to and infect neighboring cells from the hypothesis of cell-to-cell transmission. Most likely both mechanisms operate but to varying degrees. We note, however, that random seeding of the clusters from the blood, as we assumed, and the observed selective pressure of HCV specific antibodies [35], [36] suggests that transmission by freely diffusing viral particles remains an important mode of transmission throughout chronic infection. Another possible explanation for the clustered distribution of infected hepatocytes could be that some cells in initially homogeneously infected regions are cleared by innate and adaptive immune responses. While we cannot formally reject these other hypotheses, we think that the sum of our data more likely supports additional local spread.

Using mathematical models to describe the accumulation of intracellular viral RNA with time, we estimate that the hepatocytes we have analyzed have been infected for less than 7 days (Table 2 and Text S2). Although previous estimates for the turnover of infected hepatocytes based on the kinetics of plasma HCV viral load decrease under treatment have been highly variable among different patients [3], [22], [37], a large cohort study of 2100 chronically HCV infected patients under treatment with peginterferon *α*-2a with or without ribavirin estimated the average lifetime of infected hepatocytes to be around 5–7 days [21]. Several studies examined the proliferation of hepatocytes during chronic HCV infection [38]–[41]. Staining for the cell proliferation marker Ki-67 shows that between 1%–2% [38]–[40], and up to 10% (for early stages of liver inflammation as here [41]) of hepatocytes were Ki-67^+^ during chronic HCV infection. If we assume that an hepatocyte takes ∼24 h to undergo cell division *in vivo*, then these Ki-67 measurements imply 1%–10% of hepatocytes are dividing per day. We found 20%–40% of cells infected, with a turnover rate of ∼0.14/day (corresponding to a 7 day average lifetime), thus we predict an overall turnover rate in these chronically infected individuals of between 3% and 6% per day, in agreement with the estimates based on Ki-67.

Our mathematical models calculating the age of infection assumed that each hepatocyte has the same maximum number of intracellular replication complexes and the same maximum level of intracellular viral RNA, 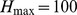 copies/cell. However, individual hepatocytes might vary substantially in their replication dynamics, possibly due to local innate and adaptive immune responses, as well as cell specific factors [42]. In addition, the maximal amount of intrahepatic HCV RNA levels could be variable. We found infected hepatocytes with up to 50 IU/cell (∼100 HCV RNA copies/cell) (Table 1); other *in situ* studies found similar numbers. For example, Chang *et al.* found a maximum of 74 HCV RNA molecules per hepatocyte [19] and Stiffler *et al*. observed from less than one copy to a maximum of 10 copies per cell [14], averaged over all cells. If one assumes that about 10% of cells are infected [12], [17], [19], then their estimate is similar to our estimates for the number of HCV genomes per infected cell. *In vitro* observations indicate much higher numbers, with cells accumulating thousands of copies of viral RNA within 72 hours [43]–[50]. However, the systems used *in vitro* typically involve high multiplicity of infection or transfection and the cells used, i.e., hepatoma cells, are highly permissive to infection, often with a very diminished or even absent endogenous interferon response, impairing the comparison to the situation in hepatocytes *in vivo*. If 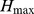 was substantial higher *in vivo* than what we observed, we would expect the infected cells in our biopsies to have been infected even more recently. Such a short lifespan seems unlikely. While it is appropriate to use the amount of intracellular HCV RNA in infected hepatocytes as a surrogate for the time since infection, without knowing all the relevant factors that might influence viral replication and accumulation (e.g. type I IFN-responses, etc.), the accuracy of estimates for the time since infection of a cell based on intracellular HCV RNA content are difficult to judge. However, less than 7 days of infection for the cells in our samples seems a consistent average estimate using different mathematical methods.

We found that the total size of a cluster did not correlate with the amount of HCV RNA in the cell that presumably founded the cluster (Figure 3A). Such correlation would be expected if cell infection continued unimpeded, because higher HCV RNA levels in the founder cell would correspond to a cell infected for longer and, hence, to a potentially longer period of cluster expansion. The biphasic decline of the viroscape of a cluster with increasing cluster extension, particularly for large clusters (Figure 3B), indicates that small sub-clusters characterized by cells with high levels of intracellular viral RNA are surrounded by larger areas of cells containing substantially lower viral load. Assuming a biphasic linear or biphasic exponential decline provided a better description of the underlying data than their monophasic analogons. A decline, although not the specific biphasic profiles that we find evidence for, has been reported before using other techniques [12], [17], [19]. Such a biphasic profile could indicate that local factors influence the progression of infection and the expansion of a cluster, such as local immune responses. One scenario could be that during establishment of the cluster, i.e. when the founding cell is infected, the viral landscape is mainly determined by the ratio between the rate of accumulation of viral RNA within a cell, including effects of the initial delay before viral RNA production starts, and the rate at which neighboring cells are infected. As infection progresses, infected cells and their neighbors start the production of antiviral factors, such as type I IFN, that protect uninfected cells from getting infected and interfere with the viral replication within infected cells. The “wave” of antiviral factors might overtake the propagation of infection, inhibiting viral production within infected cells at the border of a cluster and limiting cluster expansion. This would lead to a flatter viral landscape at the edges of a cluster. In this scenario, the first phase of decay in the amount of HCV RNA per cell with increasing cluster extension could be used to determine the ratio between viral transmission and viral replication, and the onset of the second phase might allow us to determine the effectiveness of the endogeneous antiviral response in the liver in these treatment naïve patients. Whether local infection stimulates a local immune response is still controversial [14], [15], [17]. We did not find any correlation between infection status of a cell and the expression of interferon in those cells or neighboring cells in our previous work assessing one interferon stimulating gene (ISG) [15], in agreement with other results [14]. However, a recent report did find significant co-localization of ISG expression and HCV infection [17]. More details about the influence of endogeneous type I IFN responses on the spatial propagation of HCV infection within the liver, as well as about the dynamics of cluster propagation and IFN expression, are needed to determine the extent to which these response might limit viral transmission and replication [42], [51], [52]. Such factors could also explain why a correlation between cluster size and HCV RNA content could not be observed.

At the edges of the clusters, the minimal amount of HCV RNA within cells is between 1–2 IU/cell (Figure 3B), which is close to the limit of detection. However, the possibility that the second phase of decay might be due to an artifact of the experimental method, i.e., measuring of extracellular viral RNA that sticks to the surface of the cell, is rather unlikely. Assuming that a hepatocyte has a cubic shape with a side of 20 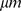, and that liver sinusoids have around the same diameter, the fluid volume above the apical surface of a cell can be approximated by a cylinder of radius 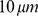 and length 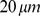, i.e., 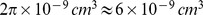. With a typical plasma viral load of 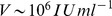, the chance that a virion would be in the fluid volume around a cell is less than 1.2%, suggesting that it is unlikely that the low HCV RNA levels at the edge of a cluster are due to extracellular virus. More precise estimates could be made if information on the rates of virus binding, dissociation and entry were available, but it seems likely that the shape of the viroscape is a characteristic of the dynamics of local cellular infection, rather than an artifact of the experimental method.

A recent study reported a significant correlation between the proportion of infected hepatocytes and serum viral load [17]. Here we presented a mechanistic dynamic model that explains this result. Indeed, the picture of local infection described above, even with the caveats discussed (low sample size, 2D sections), seems to be an appropriate and consistent representation of infection in the whole liver. In fact, we used this dynamic model of viral infection to show that the predicted serum viral load in the patients we analyzed is very similar to their measured plasma viral load, if we assume that the patterns of infection (proportion of infected cells, clustering, level of intracellular HCV RNA) observed in the biopsy samples are replicated throughout the liver.

While this result indicates the consistency of using models on ordinary differential equations (ODE) to analyze viral dynamics on a systems level, the observed spatial heterogeneity of HCV infection within the liver suggests the importance of considering space when analyzing infection dynamics within solid tissue on a cellular level. Current HCV viral dynamics models are based on infection by cell-free virus, neglecting cell-to-cell transmission or local effects of IFN responses [8], [53]. It will be interesting to incorporate these features in future models, and to examine how well these models agree with viral declines observed in patients on treatment.

We based our analysis on a small number of cells, studied in unprecedented detail, assuming that they are a reasonable representation of the infection process. Due to the estimated level of heterogeneity, more data are needed to predict the frequency of infected hepatocytes in a chronically infected patient, as well as to analyze the influence of type I IFN responses on the observed spatial patterns. Still, our method represents a novel approach to infer infection dynamics from static spatial data.

## Materials and Methods

### Patient data

In four chronically HCV infected patients (Subject 1–4) up to three sections of liver tissue were analyzed by single cell laser capture microdissection (scLCM). On each section, a grid of 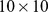 hepatocytes was analyzed for their HCV RNA content (see Fig. S1 for the individual results) as previously described [15]. The sensitivity level of the method to detect HCV RNA was set at 1 IU/cell. HCV RNA content was normalized to 7SL expression, a small ribosome-associated RNA which is abundant in the cytoplasm [15]. Using rigorous controls we ensured the precision of our method in determining single cell measurements (see *Supporting Information* Text S3). Cells in which the HCV RNA content could be detected but could not be normalized due to missing 7SL expression measurements were treated as infected throughout most of the analysis. None of the subjects had received treatment, and none were co-infected with HIV or HBV. All patients had genotype 1 infection and limited liver disease (Metavir 0–1). Full details of the experimental methods can be found in Kandathil *et al.* [15]. The experimental data are provided in the *Supporting Information* (Dataset S1).

### Characterization of clusters of infected hepatocytes

#### Spatial distribution of infected cells

We modeled the spatial distribution of infected hepatocytes in the liver as a *Matérn cluster process*, a specific type of spatial point process. A Matérn cluster process assumes that cluster centers are distributed according to a spatial Poisson process determined by the spatial intensity 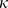, i.e., the expected number of cluster centers per unit area. In addition, it assumes regular cluster shapes with all elements of a cluster distributed in a disc with radius 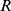 around the cluster center. The number of elements within these discs is assumed to follow a Poisson process with parameter 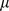 describing the expected number of elements per cluster (see also Text S1 for a more detailed description of the *Matérn cluster process*). Fitting a *Matérn cluster process* to the spatial point patterns allows us to estimate the average radius of a cluster, 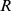, as well as the average number of cluster centers per unit area, 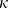 (see Text S1 for a more detailed description of this procedure).

As the Matérn cluster process assumes continuous space, and as we are also interested in the internal structure of a cluster, we determined clusters of infected cells based on the clustering of HCV RNA molecules. The following multistep-process describes the procedure of characterizing clusters of infected cells as shown in Figure 1B:

*I. Converting discrete to continuous.* In a first step, we transformed the discrete data (HCV RNA in IU/cell) into continuous data (i.e. HCV RNA IU per 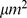): We assume that each hepatocyte has a rectangular shape of 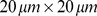, and that the 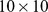 cell-grid represents a 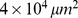 section of liver tissue. The amount of HCV RNA measured for each cell is then randomly distributed inside the allocated square for this cell in the lattice (Figure 1B). For example, if we measured 34.1 HCV RNA IU/cell, 34 HCV RNA units were positioned inside the cell at random locations, and an additional HCV RNA unit was added to the cell's content with probability 0.1. For infected hepatocytes for which the normalized HCV RNA content could not be determined, we approximated their HCV RNA content by the average over the surrounding cells. To assess the sensitivity of our results to this assumption, we performed an additional analysis setting the HCV RNA content in these cells to the detection limit of 1 IU/cell. This approach did not change our results in terms of the maximal cluster extension nor the internal cluster structure. A *Matérn cluster process* was fitted to the obtained point pattern using the package spatstat in 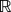, yielding an estimate of the domain radius 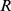, and the mean number of cluster centers, 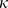. To guarantee that the particular distribution of HCV RNA inside each cell does not affect our results, we performed 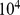 bootstrap replicates of the distribution of HCV RNA inside each cell, creating 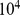 different spatial point patterns to estimate 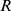 and 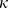.

*II. Determining the size of a cluster.* The Matérn cluster process works on a continuous spatial scale (in 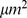) and tries to determine regions with equal densities of HCV RNA. However, infected cells vary substantially in their amount of intracellular HCV RNA. Therefore, cells that arguably belong to a cluster but have a very low amount of HCV RNA compared to other cells might not be included into the calculation of the cluster radius 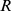, leading to an underestimation of the actual cluster size (see Figure 1B- Step I). As we want to determine the maximal size of a cluster of infected cells, hence, the maximal extension of an area of connected hepatocytes, we adjust the calculation method in order to avoid the exclusion of cells with lower densities of HCV RNA. To this end, we iteratively reduced the amount of HCV RNA inside the “core” cells of the cluster to the next measured lower level as follows: Let 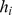, 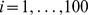 denote the amount of HCV RNA in each of the 100 measured hepatocytes in the sampled grid, with 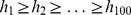. In the first step, we created a point pattern, 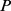, with the original data, 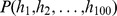, providing the domain radius 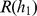 and the spatial intensity 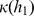 of the core of the cluster, i.e., around the cells with the highest amount of HCV RNA. In the next step, 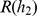 and 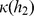 are estimated for the point pattern given by 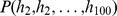, where the amount of HCV RNA in the cell with the highest amount of HCV RNA is reduced from 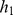 to 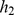. This will give us the maximal extension of a cluster containing cells with an HCV RNA content of at least 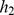. The procedure is repeated until in the final step a *Matérn cluster process* would be fitted to the point pattern given by 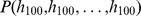. The successive reduction of HCV RNA in the cells with the highest amount to the next lower level ensures that cells have equal densities of HCV RNA, allowing the algorithm to detect larger structures (Figure 1B). For each step, 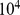 bootstrap replicates were performed where the HCV RNA for each hepatocyte is randomly distributed inside the space occupied by the cell, as described above. By this approach we define the extension of a cluster as a function of the minimum HCV RNA content in all cells assumed to belong to the cluster. Superimposing the different “rings” dependent on the minimal HCV RNA content, we obtain a “ring structure” of the total cluster of infected cells, and, hence, the distribution of cells with different amounts of HCV RNA inside the cluster.

*III. Determining maximal cluster extension.* With the algorithm described in the previous paragraph we are able to determine the radius, 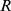, for discs of similar amounts of intracellular RNA, i.e., each cell in this disc, i.e. (sub-)cluster, has at least this amount of intracellular viral RNA. Two factors will influence the reliability of the estimate of radius 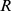 during the procedure: (1) the section of liver tissue might only show a subset of an existing cluster of infected cells, e.g., if the cluster is in a corner of the grid, and (2) not all infected cells in the analyzed grid of 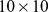 hepatocytes might belong to the same cluster of infected cells. The fitting procedure (Text S1) allows the estimation of the domain radius 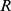 by controlling for so called edge effects, i.e., it accounts for the fact that the grid sampled by scLCM might only cover a part of the actual cluster [54], [55]. Taking edge effects into consideration allows for the estimation of cluster radii that are larger than the sampled grid of 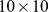 cells. However, the more 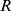 exceeds the limits of the sampled grid, the less reliable are these estimates. Using Ripley's isotropic edge correction [55], [56], estimates of 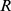 are reliable up to radii which are less than half of the diagonal of the examined region [24], [55], [56], i.e., 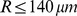 in our scenario.

As the domain radius 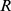 and the spatial density of cluster centers, 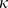, are estimated simultaneously, the fitting procedure also accounts for the fact that more than one cluster of a similar structure may be observed on the section of liver tissue. However, when iteratively adjusting the HCV RNA amount in infected hepatocytes as described in the previous paragraph, the obtained point pattern might not show signs of clustering anymore, indicating that the maximal cluster extension has been exceeded.

In order to determine the maximal extension of a cluster of infected cells, we analyzed if the spatial point pattern of HCV RNA created for each of our bootstrap replicates deviates significantly from spatial homogeneity at each step of the iterative process (see Figure 1B). To this end, we applied the Quadrant-Count-Method [57] to our point patterns (see Text S1), which is used to detect signs of spatial heterogeneity in spatial data. For this method, the examined area is divided into regular quadrants, and the number of observations, i.e., HCV RNA units, in each quadrant is counted. In our scenario, we use single cells as individual quadrants. In case of spatial homogeneity, the amount of HCV RNA per cell should be similar in each cell. Pearson's chi-squared statistic was used to determine a statistically significant deviation from spatial homogeneity (Text S1). The maximal extension of the total cluster, 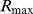, is then determined as the maximal estimated radius for which the frequency of point patterns in the 10,000 bootstrap samples deviating from spatial randomness is not smaller than 95%.

### HCV RNA content in a cell as a function of cluster extension

Based on our results on the characterization of clusters of infected cells (Fig. 3B), we found that the amount of HCV RNA in each infected hepatocyte, 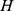, decreases with increasing cluster extension, 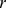. We used different models to describe this decline in HCV RNA viral load, assuming either monotonous or biphasic decay. Assuming biphasic linear decay, this decline in HCV RNA viral load can be described by the following function
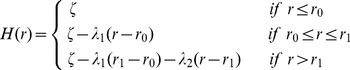
(1)


Here, the parameter 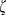 determines the maximal HCV RNA content measured in a single cell belonging to this cluster, and 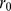 is the minimal radius of a cluster, where each cell has an HCV RNA content of 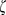 (Fig. 1A). The parameters 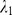 and 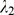 define the rates at which the HCV RNA amount within a cell is decreasing with increasing cluster size for phase 1 and 2, respectively, while 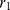 determines the cluster extension at which the rate of decrease changes. Equation (1) is fitted individually to the data of each of the different grids shown in Figure 3B, and the parameter values for 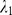, 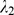 and 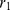 are estimated. The estimated functions for 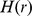 are then used to sample the HCV RNA content in cells of simulated clusters in order to determine the relationship between the total size and the total intracellular viral load of infected cells. In addition to the assumption of a biphasic linear decay, we also fitted a model assuming an exponential decay, as well as a biphasic exponential decay of intracellular HCV RNA content with increasing cluster size to the data (*not shown*). All these assumptions do not change our results with regard to the relationship between cluster size and total viral burden in the cluster. Models assuming a biphasic decay show better fits across all patients and samples judged by the mean residual sum of squares (MRSQ), i.e., the residual sum of squares divided by the difference between the number of data points and the number of free parameters, as well as the Akaike information criterion, than their monophasic analogons (mono-linear, mono-exponential).

### Determining cluster sizes based on the estimated cluster radius, 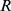


Fitting a Matérn Cluster process to the spatial point patterns of HCV molecules as described above, we are able to determine the radius, 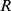, for discs of similar amounts of intracellular RNA, i.e., each cell in this disc has at least this amount of intracellular viral RNA. To get a better impression on actual cluster sizes, we convert this radius based on HCV molecules back to cell numbers. As the Matern Cluster process assumes spherical cluster shapes and HCV RNA molecules are distributed randomly within each cell (size  = 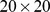 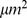), the obtained radius might cut only a part of a cell, or include parts of uninfected cells (see Fig. 1B). Therefore, we calculated a minimal and maximal cluster size in number of cells, assuming either a radial or quadratic cluster extension: The minimal number of cells belonging to a disc with radius 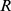 is determined by 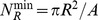, where 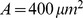 denotes the area of a hepatocyte. The possible maximal number of cells in a disc is estimated by assuming the area of the cluster is given by a square with edge length 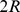, hence, 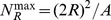.

Our stepwise algorithm determines the cluster extension, 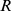, as a function of the minimal amount of intracellular HCV RNA, 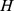, in a cell belonging to a certain (sub-)cluster. By piling the different discs on top of each other (Fig. 1B), the total clusters are assumed to be structured like the rings in a tree trunk with each “cell-ring” around the founder cell having similar intracellular HCV RNA content. When reconstructing individual clusters in terms of cluster size and intracellular viral RNA, we have to determine the number of cells and the range of the HCV RNA content in cells belonging to a ring at a fixed distance to the founder cell of the cluster. Therefore, for each bootstrap replicate, we sampled the number of cells, 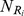, belonging to a disc with radius 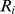, 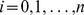, with 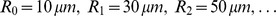, based on a uniform distribution on 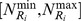. The actual number of cells in each ring 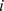 is then defined by 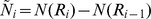, with 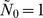, i.e., the assumed founder cell. In addition, each of the infected hepatocytes of ring 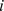 was filled with an amount of HCV RNA 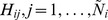 uniformly sampled from 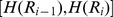, the range of the amount of HCV RNA estimated in the corresponding ring. The total number of cells belonging to the cluster, 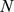, as well as the total HCV RNA content in the cluster, 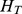, was then calculated based on the sum over all “rings” of the cluster, hence, 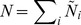, and 
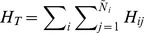
. For each cluster, 10,000 bootstrap replicates were performed.

### Age of infection

We use a stochastic model of HCV replication dynamics developed recently [27] to estimate the age of infection of a cell based on its amount of intracellular HCV RNA. In brief, after an initial time 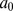 post infection, replication complexes are formed over an average time of 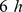 based on *in vitro* experimental data where negative strand RNA, assumed to represent replication complexes, is first detected 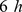 post infection [50]. Each replication complex generates a new replication complex with a probability of 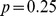 per generation time of 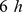 [27]. Positive strand viral RNA is produced by replication complexes with on average 75% of the newly generated viral RNA estimated to be exported in virions [27]. The remaining viral RNA accumulates within the cell, increasing the intracellular amount of positive strand HCV RNA. In our data, we observe a maximal intracellular HCV RNA amount of 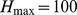 HCV RNA copies/cell, corresponding to the observed level of up to 50 IU/cell (1 IU 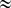 1.96 genome copies [15]), which is consistent with other studies [14]. For each cell, we run 10,000 instances of the stochastic model of viral replication and formation of replication complexes, varying 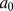 between 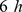 and 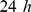 [50] to estimate the age of infection based on the amount of intracellular HCV RNA. In addition to the stochastic model, we also analyze the data using an analytical model as developed in [5] (see Text S2).

### Comparing intracellular viral RNA and viral load in plasma

We used an age-structured mathematical model [9] to compare the intracellular HCV RNA content per hepatocyte (in up to 300 hepatocytes analyzed per patient) to the total serum viral load. This model explicitly describes the HCV viral load, 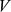, as a function of the intracellular HCV RNA content of single hepatocytes, 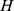. The model equation for 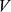 is [9], [10]:
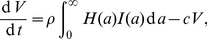
(2)where the intracellular amount of viral RNA in an hepatocyte, 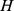, is dependent on the time since infection, 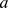, called the age of infection of a cell. Intracellular HCV RNA is packaged into virions and exported from an infected cell at rate 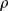. The extracellular viral load, 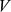, increases due to the HCV RNA export at rate 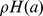, from each infected hepatocyte of age 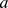, 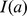, and decreases due to viral clearance at rate 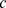 per virion. For each patient, the viral load measured in HCV RNA copies per 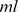 (or international units (IU) to normalize among HCV RNA assays) of serum was determined at the time of biopsy (see Table 3). As each patient was in the chronic stage of HCV infection, we assumed that viral load had reached steady-state, 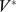. According to Eq. (2), the measured viral load, 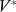, would be related to the total number of infected hepatocytes in the liver, 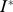, by
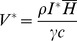
(3)where 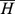 denotes the average HCV RNA content in an infected hepatocyte. The parameter 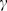 is a scaling factor to account for the fact that 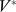 is measured per ml of serum, while 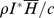 defines the total number of HCV viral particles in the human body. This scaling factor is set to 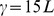, the average total extracellular fluid volume for a 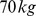 individual [58]. To compare the measured serum viral load, 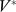, to the individual liver sections, we estimate the viral load produced by 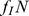 infected cells, where 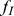 denotes the estimated frequency of infected hepatocytes for each subject, and 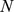 the total number of hepatocytes in a human liver, hence, 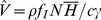. If 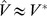, the inferred dynamics from the sampled liver sections are consistent with the average infection dynamics in the whole liver.

To calculate 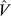, we first determined the frequency of infected hepatocytes in the individual liver samples, 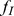, as well as the observed mean HCV RNA content in infected hepatocytes, 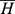, combining all sections of one subject (see Table 3). The total number of hepatocytes in a human liver is in the range of 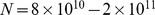 cells [28]–[31], and we used the upper limit of 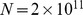. The viral clearance rate 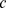 and the viral export rate for HCV, 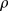, have been estimated recently as 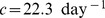 and 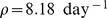 [10].

### Statistical analysis

The statistical dependency between different attributes of clusters of infected cells (e.g. size of cluster and total amount of HCV RNA) was analysed using Spearman's correlation coefficient and linear mixed effects models, with subject as the random effect. Linear mixed effects models take into account that several sections of liver tissue originate from the same patient. All analyses were performed using the 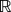 language of statistical computing [59]. Core functions are provided in the Supporting Information (Protocol S1).

## Supporting Information

Figure S1
**Measured HCV RNA content per patient.** For each patient, the HCV RNA content per hepatocyte measured by single cell laser capture microdisection (scLCM) is given in IU/cell. The sensitivity level of the method was 1 IU/cell. Grey boxes indicate infected hepatocytes for which the normalized HCV RNA content could not be determined. Their intracellular HCV RNA amount is approximated according to different methods (see *Materials & Methods*). A possible distribution of clusters according to the determined cluster sizes is sketched as well. Please note that the estimated cluster radius for grid 1 of subject 1 has to be taken with care. Here, the cluster detection algorithm seems to be affected by the distribution of infected cells as it determines clusters of radial shape (compare also to Figure S2 where the estimate of 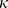 is increasing after the cut-off criterion in contrast to expectation, and compared to all other grids).(TIF)

Figure S2
**Estimates of the domain radius** 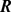 **, subject 1.** Estimates of the domain radius 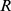 (**A**) and the spatial intensity 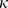 (**B**) dependent on the minimal HCV RNA content for cells assumed to form a cluster for each of the three different grids on the sections of subject 1. Plots should be read from the right to the left as the algorithm starts at point 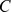, the maximal amount of HCV RNA measured in an infected cell on the indicated slide. In (**A**), the domain radius of the cluster, 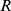, is given on a continuous scale, as well as in number of cells. The red line gives the median over 10,000 bootstrap replicates of fitting a *Matérn cluster process* to the data as described in *Materials & Methods*. The red area denotes the 95%-quantiles of the estimates. The dashed horizontal (**A**) and vertical (**B**) lines indicate the cutoff of the algorithm, i.e., the maximal extension of the total cluster.(TIF)

Figure S3
**Estimates of the domain radius** 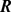 **, subject 2.** For details see explanation under Figure S2.(TIF)

Figure S4
**Estimates of the domain radius** 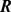 **, subject 4.** For details see explanation under Figure S2.(TIF)

Text S1
**Details on the clustering analysis.** Detailed explanations on the Matérn cluster process, the estimation of the domain radius, 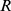, and the Quadrant-Count method.(PDF)

Text S2 **Alternative model to determine age of infection.** Estimated age of infection of cells based on the amount of intracellular HCV RNA using an alternative model. The model and the obtained estimates are explained in detail.
(PDF)

Text S3 **Details on simulation and experimental methods.** Details on the simulation method to simulate cluster expansion and extended discussion of the experimental method.
(PDF)

Dataset S1 **ExperimentalData.xls.** Excel sheet with the experimental data of the liver biopsy samples of the 4 subjects analyzed by scLCM [15].
(XLS)

Protocol S1
**MaternClustering.R.** 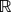-code with the core functions used for analyzing the data with a Matérn Cluster process as shown in Figure 1B.(R)
