# Supplementary figures and images for: Inferring Viral Dynamics in Chronically HCV Infected Patients from the Spatial Distribution of Infected Hepatocytes
Source: PLoS Comput Biol. 2014 Nov 13;10(11):e1003934. doi: 10.1371/journal.pcbi.1003934 (PMC4230741; doi:10.1371/journal.pcbi.1003934)

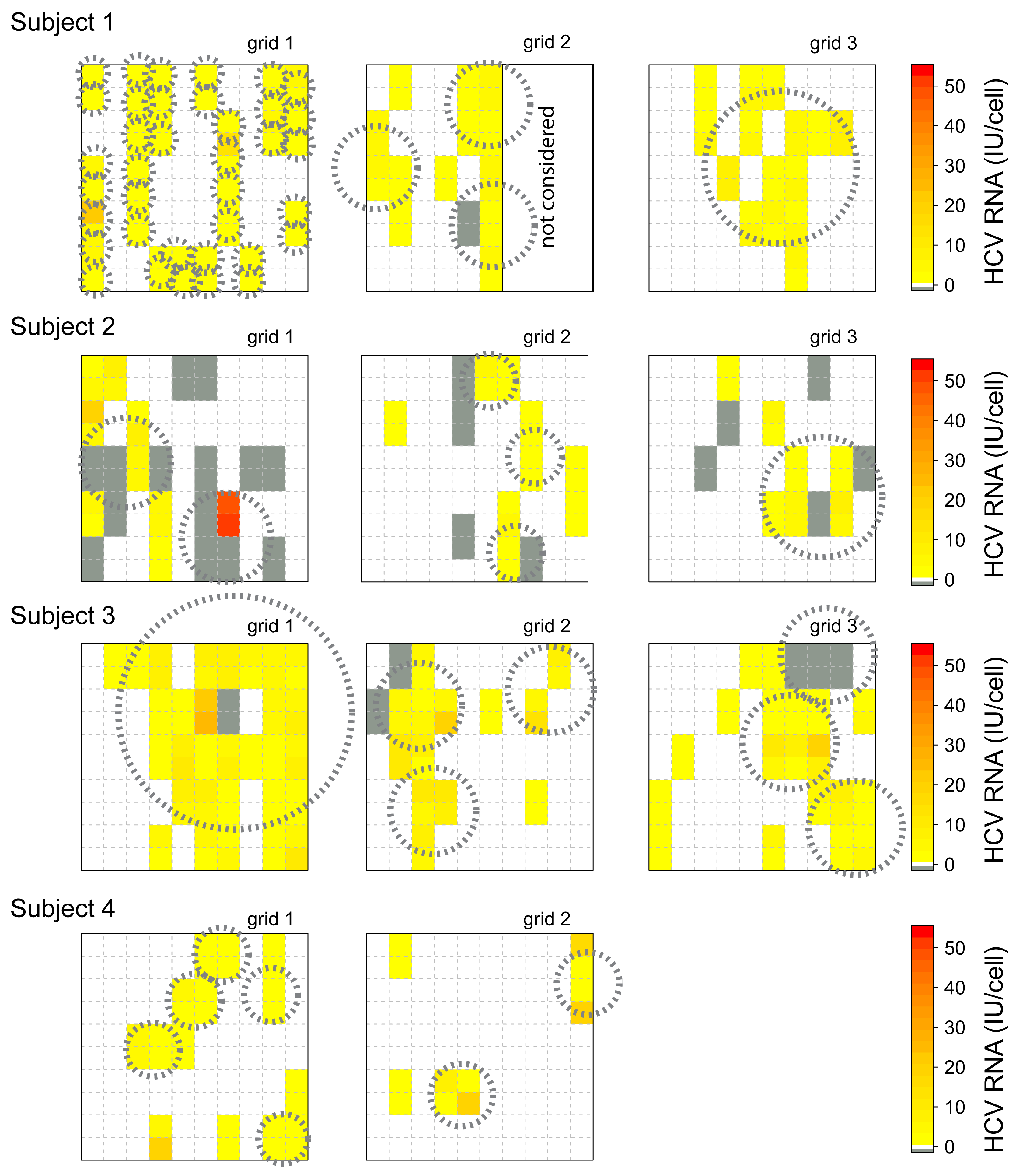

Supplement: Figure S1 — Measured HCV RNA content per patient. For each patient, the HCV RNA content per hepatocyte measured by single cell laser capture microdisection (scLCM) is given in IU/cell. The sensitivity level of the method was 1 IU/cell. Grey boxes indicate infected hepatocytes for which the normalized HCV RNA content could not be determined. Their intracellular HCV RNA amount is approximated according to different methods (see Materials & Methods). A possible distribution of clusters according to the determined cluster sizes is sketched as well. Please note that the estimated cluster radius for grid 1 of subject 1 has to be taken with care. Here, the cluster detection algorithm seems to be affected by the distribution of infected cells as it determines clusters of radial shape (compare also to Figure S2 where the estimate of is increasing after the cut-off criterion in contrast to expectation, and compared to all other grids). (TIF) [file pcbi.1003934.s001.tif]

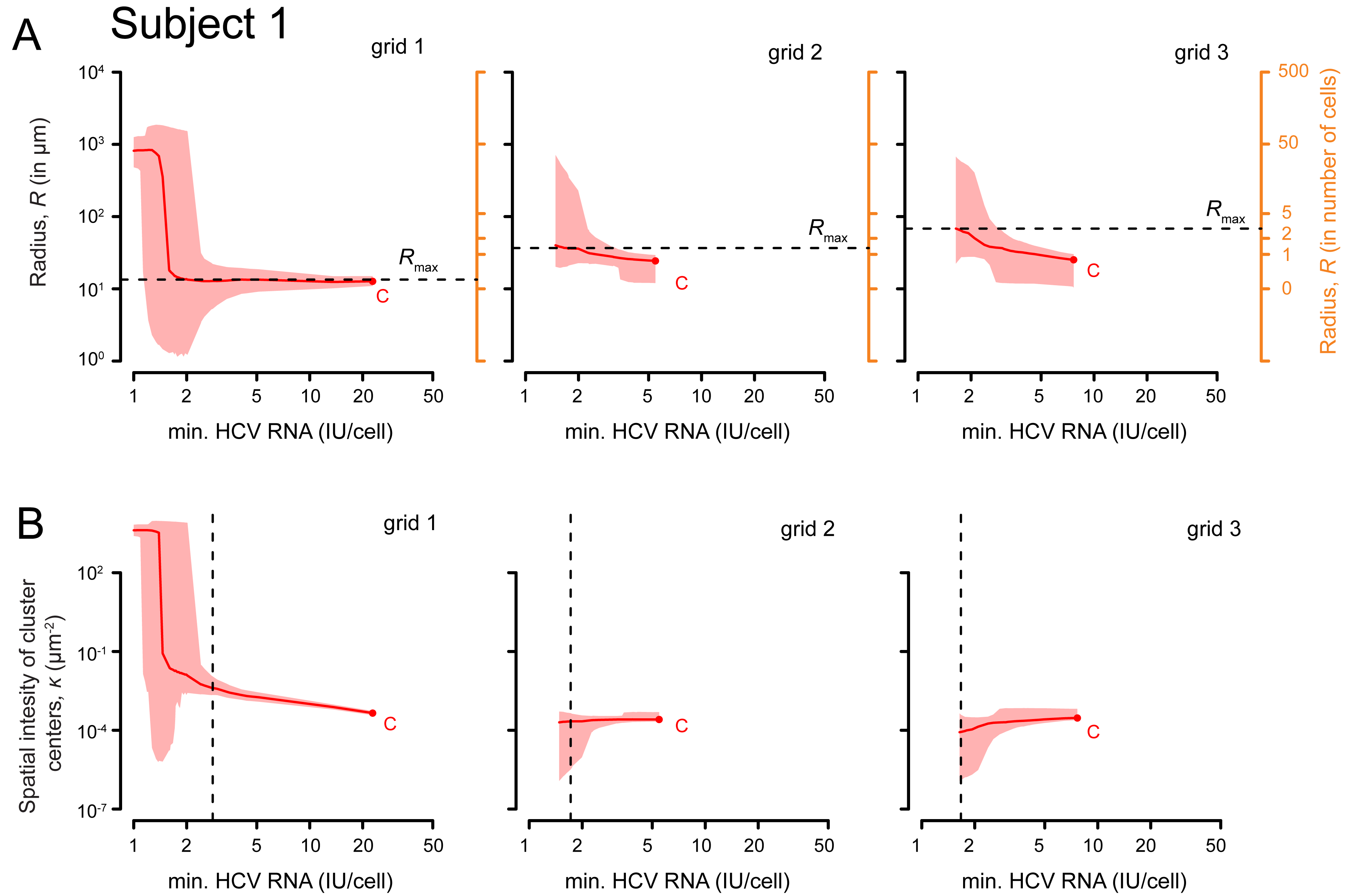

Supplement: Figure S2 — Estimates of the domain radius , subject 1. Estimates of the domain radius (A) and the spatial intensity (B) dependent on the minimal HCV RNA content for cells assumed to form a cluster for each of the three different grids on the sections of subject 1. Plots should be read from the right to the left as the algorithm starts at point , the maximal amount of HCV RNA measured in an infected cell on the indicated slide. In (A), the domain radius of the cluster, , is given on a continuous scale, as well as in number of cells. The red line gives the median over 10,000 bootstrap replicates of fitting a Matérn cluster process to the data as described in Materials & Methods. The red area denotes the 95%-quantiles of the estimates. The dashed horizontal (A) and vertical (B) lines indicate the cutoff of the algorithm, i.e., the maximal extension of the total cluster. (TIF) [file pcbi.1003934.s002.tif]

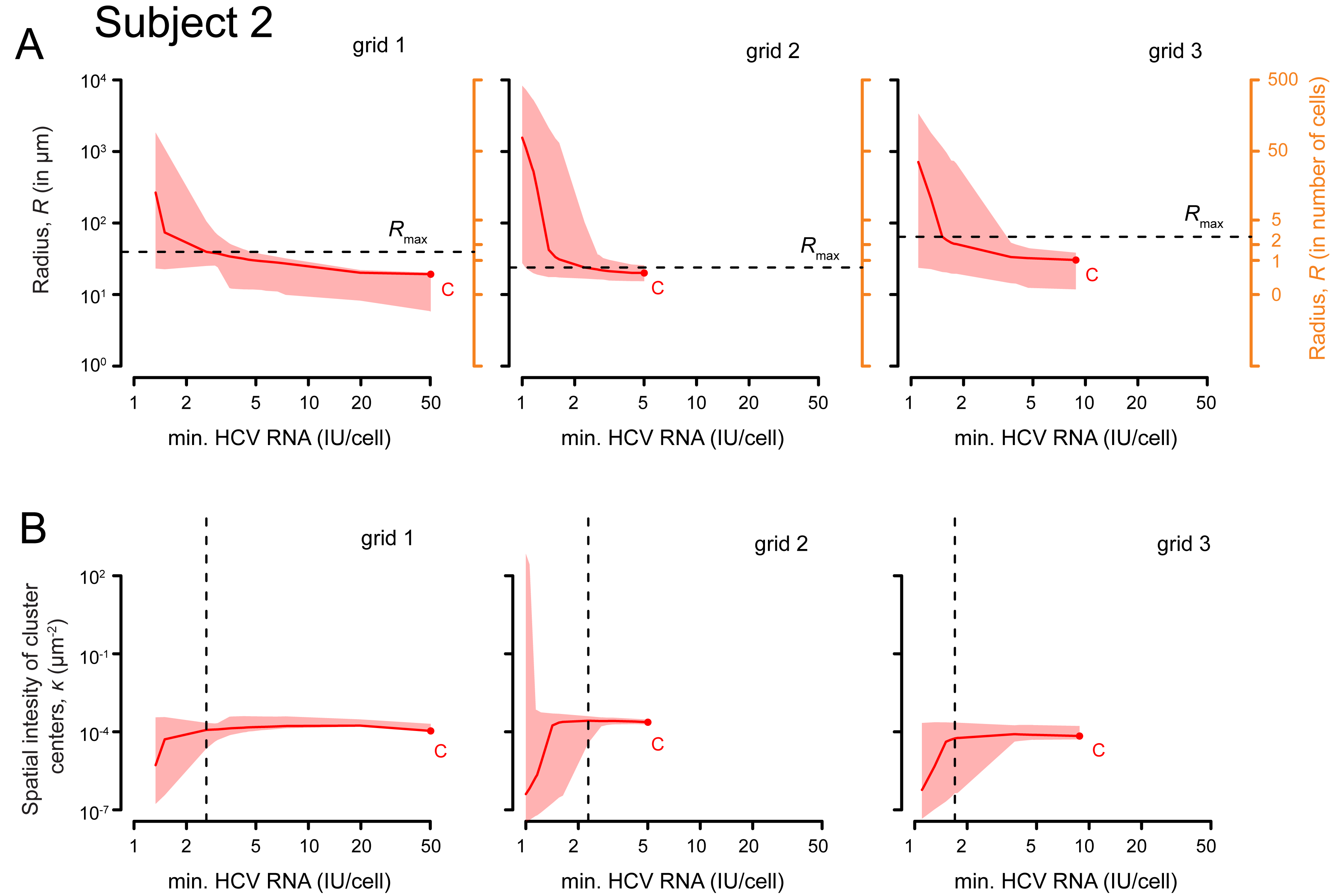

Supplement: Figure S3 — Estimates of the domain radius , subject 2. For details see explanation under Figure S2. (TIF) [file pcbi.1003934.s003.tif]

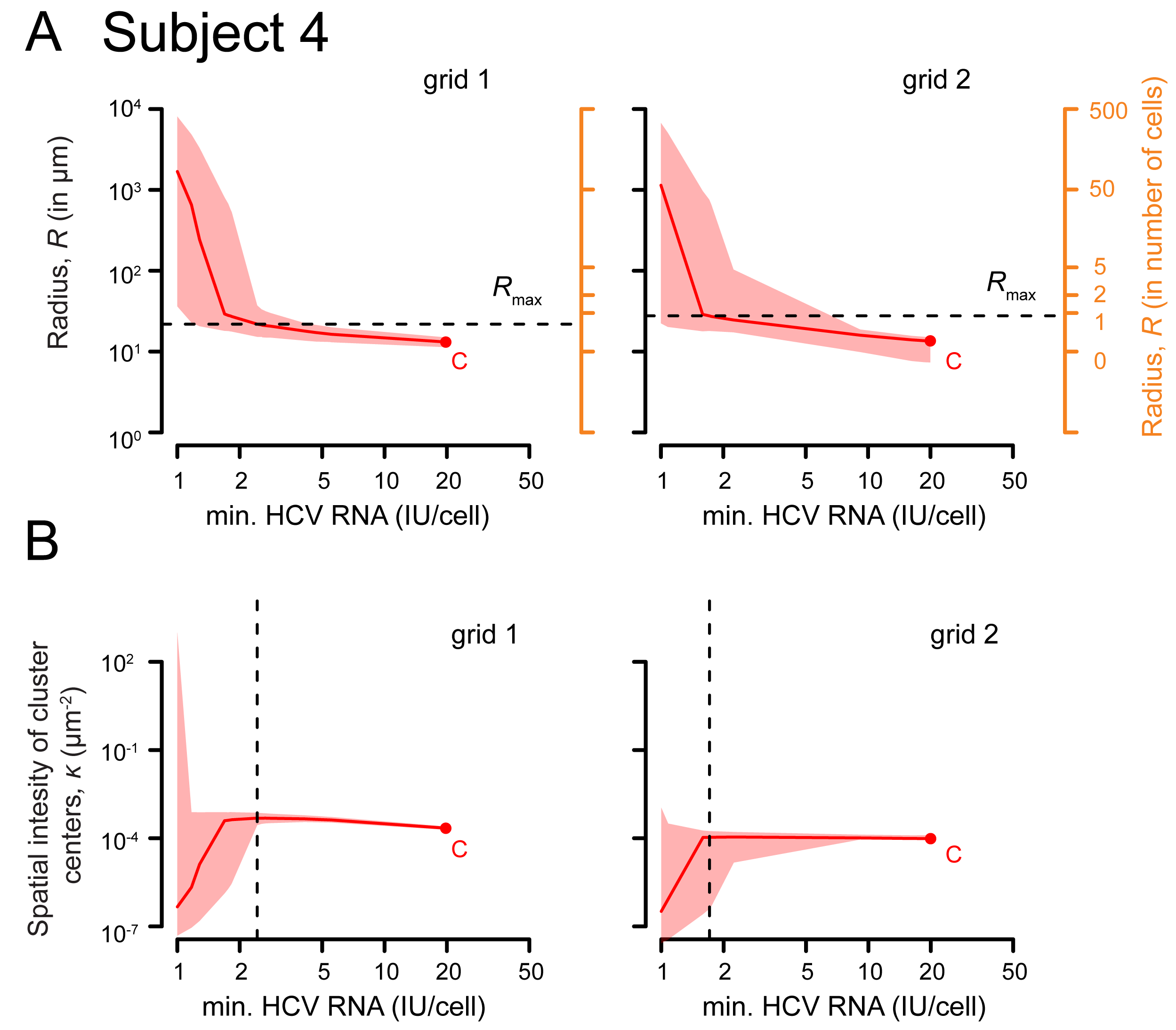

Supplement: Figure S4 — Estimates of the domain radius , subject 4. For details see explanation under Figure S2. (TIF) [file pcbi.1003934.s004.tif]
