# Supplementary material for: Inferring Viral Dynamics in Chronically HCV Infected Patients from the Spatial Distribution of Infected Hepatocytes
Source: PLoS Comput Biol. 2014 Nov 13;10(11):e1003934. doi: 10.1371/journal.pcbi.1003934 (PMC4230741; doi:10.1371/journal.pcbi.1003934)
Supplement: Text S1 — Details on the clustering analysis. Detailed explanations on the Matérn cluster process, the estimation of the domain radius, , and the Quadrant-Count method. (PDF) [file pcbi.1003934.s005.pdf]

S1 Details on the clustering analysis

S1.1 The Matérn cluster process

In mathematical terms, we can describe the distribution of infected cells in the liver as a spatial point process [1, 2]. Each infected hepatocyte, Y_i, $i = 1, \dots, n$ is the realization of a certain spatial distribution. Let $S \subset \mathbb{R}^2$ denote a 2 dimensional section of liver tissue. We assume that the initiation of foci of infected cells in the liver, the seeding of clusters, occurs randomly following a spatial Poisson process. This means for every closed set of cells $B \subset S$, the number of cluster centers in this subset, $N_C(B)$ follows a Poisson process with mean $\kappa A(B)$, where $\kappa > 0$ determines the uniform intensity of a Poisson process for the cluster centers and $A(B)$ the area of $B \subset S$. This means

$$N_C(B) \sim \text{Poisson}\left(\kappa A(B)\right) \tag{1}$$

The intensity κ defines the expected number of cluster centers per unit area. Each of these randomly distributed cluster centers gives rise to a cluster of infected hepatocytes. As expected for randomly distributed count data, we assume that the number of infected hepatocytes per cluster also follows a Poisson distribution with expectation μ. In general, each cluster is assumed not to exceed a disk with radius R, the domain radius, around the cluster center. In total, infected hepatocytes are assumed to follow a spatial distribution with intensity $\lambda = \kappa\mu$. This kind of spatial distribution is explicitly defined as the so called Matérn cluster process [1]. See Figure A1 for two realizations of a Matérn cluster process.

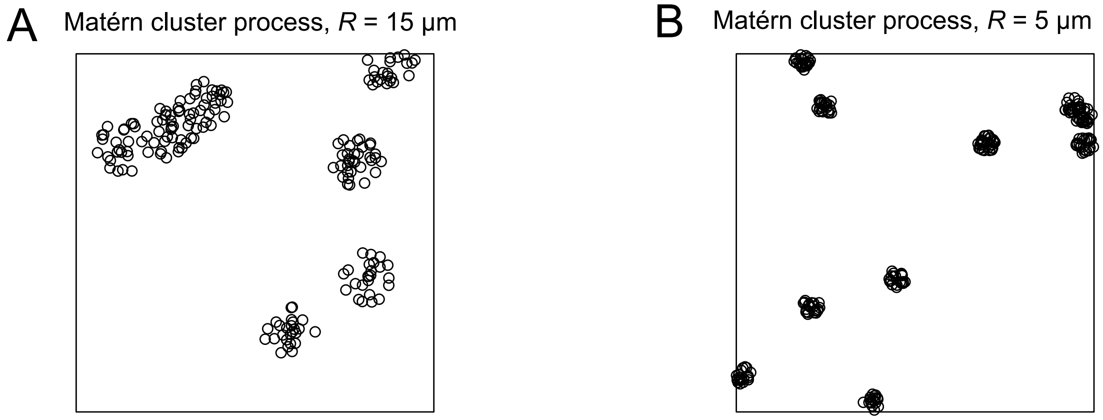

Figure A1: Realizations of a Matérn cluster process with an intensity of cluster centers of $\kappa = 0.02$ per $100\,\mu m^2$, and the number of dots per cluster following a Poisson distribution with expectation $\mu = 25$. The radius of cluster areas, e.g. the domain radius, is defined as $R = 15\,\mu m$ (a) and $R = 5\,\mu m$ (b). Plots are shown for a region of $200\,\mu m \times 200\,\mu m$.

S1.2 Determining the domain radius R (Text S3)

The domain radius R, i.e. the average size of a cluster, is an important measurement to characterize the spatial distribution of infected hepatocytes in the liver. Combining this quantity with measurements of the time since infection can be used to estimate the rate of local spreading of infection. A method to determine the domain radius is given by Ripley's K-function [3]. In order to use this method, we transformed the discrete infected cell count into a continuous distribution of intracellular HCV RNA molecules by randomly distributing the measured intracellular amount of HCV RNA over the space in the grid occupied by this cell (see Fig. 1 in the manuscript). Let $i = 1, \ldots, m$ denote the ith HCV RNA molecule in the sample and $M_i(r)$ the observed number of HCV RNA molecules in a disk with radius r around molecule i. The second moment property of a spatial point process, in this case given by the spatial distribution of HCV RNA molecules, is the expected number of HCV RNA molecules within a distance r of a randomly chosen HCV RNA molecule. Ripley's K-function, $K(r)$ is defined as the second moment property normalized by the intensity, which is the number of points per area, λ [4]. Ripley's K-function, $K(r)$, is then defined by

$$K(r) = \frac{1}{m} \sum_{i=1}^{m} \frac{M_i(r)}{\lambda} \tag{2}$$

For a random Poisson distribution, meaning spatial homogeneity, $K(r) = \pi r^2$. To better judge deviations of the examined spatial point pattern from spatial homogeneity, $K(r)$ is usually transformed to $H(r)$ [5], with

$$H(r) = \sqrt{\frac{K(r)}{\pi}} - r \tag{3}$$

Kiskowski et al. [4] showed, that the radius R, which minimizes the derivative of H, $H'(R) = \min_r H'(r)$, is a good estimate of the domain radius. However, for experimental data, the radius r that maximizes $H(r)$, indicating the radius of maximal aggregation, should be preferred as a measurement for the domain radius R [4]. Several correction methods for the calculation of $K(r)$ are available to account for edge effects of the sampled region [6–8]. We use Ripley's isotropic edge correction for the estimation of the domain radius R, which is valid up to radii that are less than half of the diagonal of the sampled region [8], i.e., $R \leq 140\,\mu m$ in our scenario. All calculations and fitting procedures were performed using the package `spatstat` in the $\mathbb{R}$ language of statistical computing [9].

S1.3 The Quadrant-Count-Method - Pearson's chi-squared statistic (Text S4)

The Quadrant-Count-Method is used to determine if the spatial distribution of observations in a given area shows signs of spatial heterogeneity, i.e., clustering. To this end, the examined area is divided into equally sized regions (=quadrants) and the number of observations per region are counted [10]. Let $i = 1, \ldots, n$ denote the different quadrants in the examined area with N_i denoting the number of observations, i.e. infected cells or HCV RNA, in quadrant i. $\bar{N} = 1/n \sum_{i=1}^{n} N_i$ denotes the mean of the counts over all quadrants. We then calculate the following test-statistic:

$$Q = \sum_i \frac{\left(N_i - \bar{N}\right)^2}{\bar{N}} \tag{4}$$

Under the null-hypothesis that all quadrants have the same number of observations (uniform distribution), Q follows a chi-squared distribution with $n-1$ degrees of freedom. Large values of Q in comparison to a chi-squared distribution with $n-1$ degrees of freedom indicate heterogeneity and, hence, clustering.

References

[1] Baddeley A 2007. Spatial Point Processes and their Applications. Lecture Notes in Mathematics 1892: 1–75.

[2] Ripley BD 1981. Spatial Statistics. New York: John Wiley & Sons.

[3] Ripley BD 1977. Modeling spatial patterns. J R Stat Soc Series B Stat Methodol 39: 172-192.

[4] Kiskowski MA, Hancock JF, Kenworthy AK 2009. On the use of Ripley's K-function and its derivatives to analyze domain size. Biophys J 97: 1095–1103.

[5] Ehrlich M, Boll W, Van Oijen A, Hariharan R, Chandran K, Nibert ML, Kirchhausen T 2004. Endocytosis by random initiation and stabilization of clathrin-coated pits. Cell 118: 591–605.

[6] Goreaud F, Pélissier R 1999. On explicit formulas of edge effect correction for Ripley's K-function. J Veg Sci 10: 433–438.

[7] Dixon PM 2002. Ripley's K function. In: El-Shaarawi AH, Piegorsch WW, editors, Encyclopedia of Environments, Chichester: John Wiley & Sons, Ltd. pp. 1796–1803.

[8] Ripley BD 1976. The second-order analysis of stationary point processes. J Appl Probab 13: 255–266.

[9] R Development Core Team 2006. R: A Language and Environment for Statistical Computing. Vienna, Austria: R Foundation for Statistical Computing.

[10] Elliott P, Wakefield J, Best N, Briggs D 2000. Spatial Epidemiology - Methods and Applications. Oxford: Oxford University Press.
