# Supplementary material for: Inferring Viral Dynamics in Chronically HCV Infected Patients from the Spatial Distribution of Infected Hepatocytes
Source: PLoS Comput Biol. 2014 Nov 13;10(11):e1003934. doi: 10.1371/journal.pcbi.1003934 (PMC4230741; doi:10.1371/journal.pcbi.1003934)
Supplement: Text S2 — Alternative model to determine age of infection. Estimated age of infection of cells based on the amount of intracellular HCV RNA using an alternative model. The model and the obtained estimates are explained in detail. (PDF) [file pcbi.1003934.s006.pdf]

S2 Alternative model to determine the age of infection based on the amount of intracellular HCV RNA

In the main text, we used a simple model for the accumulation of HCV RNA inside an infected hepatocyte. Here we show that using a more detailed model as introduced in [1] does not change our conclusions. Let H again denote the number of intracellular positive strand HCV RNA molecules available for translation or viral assembly. Positive strand RNA serves as a template for replication complexes, U, which contain negative strand RNA and proteins, and are built at a maximum rate β per positive strand of HCV RNA. The replication complexes then synthesize and create new complementary positive strand RNA at a rate α per replication complex. Positive strand RNA is transported out of the cell at a rate ρ and gets degraded with rate μ. Replication complexes are assumed to be lost with rate γ. In addition, the model assumes that each hepatocyte can only harbor a limited number of replication complexes, $U_{\max}$. The model is then formulated by

$$\frac{\mathrm{d}H}{\mathrm{d}a} = \alpha U - (\mu + \rho) H \tag{1a}$$

$$\frac{\mathrm{d}U}{\mathrm{d}a} = \beta \left(1 - \frac{U}{U_{\max}}\right) H - \gamma U \tag{1b}$$

where a denotes the time since infection of the cell. The steady states of this system are given by

$$H^* = \frac{\alpha U^*}{\mu + \rho} = U_{\max} \left(\frac{\alpha}{\mu + \rho} - \frac{\gamma}{\beta}\right) \tag{2a}$$

$$U^* = U_{\max} \left(1 - \frac{\gamma(\mu + \rho)}{\alpha\beta}\right) \tag{2b}$$

In *in vitro* experiments and *in vivo* under type I IFN therapy, the degradation rate of intracellular positive strand RNA was estimated to be around $\mu = 1.46\,\mathrm{day}^{-1}\,(0.75, 2.17)$ [1]. The half-life of negative strand RNA, hence, the half-life of replication complexes has been estimated to be ~ 12 h *in vitro* [2] leading to a degradation rate of $\gamma = 1.39\,\mathrm{day}^{-1}$. Other studies use a degradation rate of $\gamma = 0.5\,\mathrm{day}^{-1}$ *in vivo* [1]. The rate at which HCV virions are released from the cell was recently estimated to be $\rho = 8.18\,\mathrm{day}^{-1}\,(4.65, 11.71)$ [3]. Experimental systems have not allowed a reliable estimation of the production rates of positive strand viral RNA, α, and replication complexes, β, so far.

Determining the production rate of viral RNA, α, and replication complexes, β: In the following, we will determine and constrain estimates for the production rate of viral RNA, α, and replication complexes, β. Quinkert et al. [4] measured the amount of positive and negative strand HCV RNA in a replicon system and found that there were ~ 200 positive strands and ~ 40 negative strands at steady state. Using Eq. (2a), and taking the number of negative strand RNA molecules as a proxy for the number of replication complexes inside a cell, we obtain

$$\alpha = \frac{H^*(\mu + \rho)}{U^*} \tag{3}$$

With Eq. (2b), and as $0 \leq U^* \leq U_{\max}$, we obtain a lower bound for the value of β given by

$$\beta \geq \frac{\gamma(\mu+\rho)}{\alpha} \tag{4}$$

In order to also obtain an upper bound for β, we simplified the model system given in Eqs. (1a) and (1b), only examining the eclipse phase, the time it takes for an infected cell to start releasing infectious virus. In this phase, no viral RNA gets exported, i.e. $\rho = 0$, and we assume $U/U_{\max} \ll 1$. Therefore, Eqs. (1a) and (1b) reduce to

$$\frac{\mathrm{d}H}{\mathrm{d}a} = \alpha U - \mu H \tag{5a}$$

$$\frac{\mathrm{d}U}{\mathrm{d}a} = \beta H - \gamma U \tag{5b}$$

This system of ordinary differential equations can be solved, leading to the explicit solutions

$$H(a) = \frac{(\lambda_1+\mu)e^{\lambda_2 a} + (\lambda_1+\gamma)e^{\lambda_1 a}}{2\lambda_1+\mu+\gamma} \tag{6a}$$

$$U(a) = \frac{\beta(e^{\lambda_1 a} - e^{\lambda_2 a})}{2\lambda_1+\mu+\gamma} \tag{6b}$$

where λ_1 and λ_2 define the eigenvalues of the system given in Eqs. (5a) and (5b)

$$\lambda_{1/2} = \frac{-(\mu+\gamma)}{2} \pm \frac{\sqrt{(\mu-\gamma)^2 + 4\alpha\beta}}{2} \tag{7}$$

During the eclipse phase of length τ, no viral RNA gets exported. Therefore, the production rate of replication complexes β should fulfill the following equation

$$E(\tau) = \rho\left(\int_0^\tau H(s;\beta)\mathrm{d}s - 1\right) \leq 1 \qquad , \tag{8}$$

where $E(\tau)$ denotes the number of viral RNA exported until time τ. Using Eqs. (6a) and (7) in Eq. (8), we obtain a higher bound for the production rate of replication complexes, β. Hence, with $\mu = 1.46\,\mathrm{day}^{-1}$, $\rho = 8.18\,\mathrm{day}^{-1}$, $\gamma = 0.5\,\mathrm{day}^{-1}$, and $\tau = 12\,h$, we obtain a production rate of viral RNA per replication complex of $\alpha = 24.1\,\mathrm{day}^{-1}$ and a production rate of replication complexes constrained by $0.2\,\mathrm{day}^{-1} \leq \beta \leq 1.47\,\mathrm{day}^{-1}$.

With this extended model (Eqs. (1a) and (1b)), we determined the age of infection for each of the different hepatocytes on each grid in the following way: We simulated the accumulation of intracellular viral RNA, H, with the age of infection according to Eqs. (1a) and (1b) sampling the parameters $\mu \in [0.75\,\mathrm{day}^{-1}, 2.17\,\mathrm{day}^{-1}]$ [5], $\rho \in [4.65\,\mathrm{day}^{-1}, 11.71\,\mathrm{day}^{-1}]$ [3], and $\gamma \in [0.5\,\mathrm{day}^{-1}, 1.385\,\mathrm{day}^{-1}]$ [1, 2] uniformly in these ranges. With $H^* = 100$ positive strand viral RNA per cell and $U^* = 40$ negative strand RNA per cell [4], α was calculated based on Eq. (3) and β uniformly sampled from

its constrained interval given by Eqs. (4) and (8). Using these parameters in Eqs. (1a) and (1b) to simulate the progression of intracellular viral RNA, H, and replication complexes, U, we determined the age of infection of each hepatocyte as the time point at which H equals the observed amount of HCV viral RNA. For each of the different hepatocytes, 10,000 bootstrap replicates sampling μ, ρ, γ, and β were performed. Table A2 shows the median and the 2.5% and 97.5% percentiles for the cell with the oldest age of infection in the cluster that presumably founded the cluster (age core), and the age of infection of cells in the periphery (age periphery), as well as the difference between these two values, Δ_{age}. As before, in our analysis, we concentrated on the oldest cluster on a grid, i.e. the cluster containing the cell with the highest amount of intracellular HCV RNA. As with the basic model presented in the manuscript, clusters of infected cells are estimated to be on average not older than ~ 7 days, and that the time between the infection of the founder cell and the spread to the current cluster extension is less than 4 days (Table A2).

Table A2: Age of infection of hepatocytes: For each of the different subjects and grids, the age of infection of the cell that presumably founded the oldest cluster on the grid (age core), i.e. cell with the highest amount of intracellular HCV RNA, and of cells in the periphery (age periphery) is determined according to Eqs. (1a) and (1b) that are parameterized as explained in the text with an ecplise phase of $\tau = 12\,h$. In addition, we also calculated the age difference, Δ_{age}, between these two values, indicating the speed of infection to the predicted cluster extension. The previously determined maximal cluster extension, $R_{\max}$, is indicated as well. The mean based on 10,000 bootstrap replicates is shown. Numbers in brackets represent the 2.5% and 97.5% percentiles.

subject	grid	$\mathbf{R}_{\max}$ (in μm)		age core (in days)		age periphery (in days)		$\mathbf{\Delta}_{\text{age}}$ (in days)	
1	1	12.9	(5.2,22.1)	6.26	(2.0,17.5)	4.31	(1.1,15.3)	2.71	(0.9,7.2)
	2	36.7	(20.3,407.1)	4.90	(1.3,16.0)	3.90	(0.9,15.0)	1.40	(0.4,4.3)
	3	67.3	(23.6,642.3)	5.19	(1.5,16.2)	3.87	(0.9,15.1)	1.84	(0.6,5.5)
2	1	39.5	(25.1,105.7)	8.62	(3.4,18.6)	4.23	(1.0,15.2)	6.21	(2.2,12.6)
	2	23.9	(16.7,101.9)	4.81	(1.3,15.8)	4.13	(1.0,15.3)	0.95	(0.3,3.0)
	3	53.1	(19.6,747.2)	5.31	(1.5,16.4)	3.89	(0.9,15.0)	1.99	(0.6,5.8)
3	1	103.0	(12.0,514.0)	6.45	(2.1,17.5)	4.43	(1.1,15.3)	2.75	(0.9,7.2)
	2	37.9	(20.9,389.5)	3.41	(2.0,17.3)	3.91	(0.9,15.1)	3.03	(1.0,8.2)
	3	41.5	(26.0,266.8)	4.86	(2.0,17.4)	4.20	(1.0,15.3)	2.70	(0.9,7.3)
4	1	21.8	(15.2,37.2)	6.10	(1.9,17.3)	4.18	(1.0,15.4)	2.65	(0.8,7.2)
	2	27.7	(18.0,758.5)	6.11	(1.9,17.4)	3.89	(0.9,15.0)	3.07	(1.0,8.3)

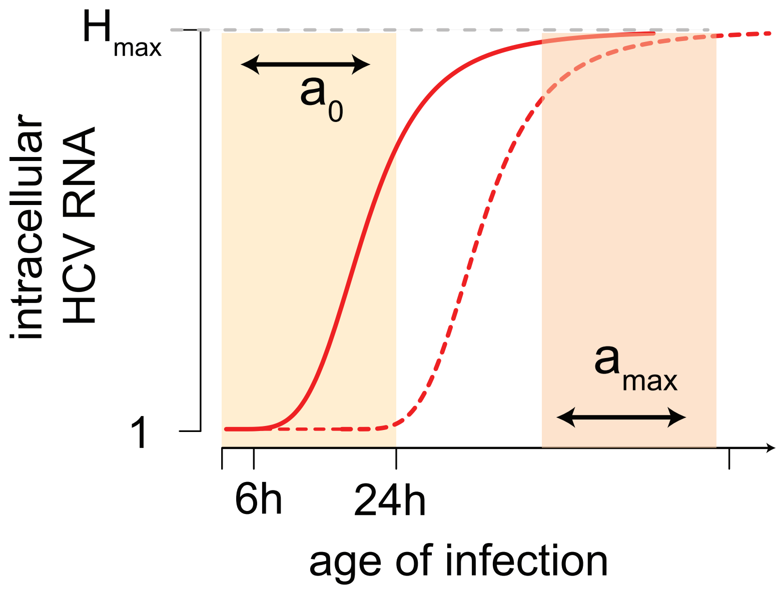

Figure A2: Accumulation of intracellular HCV RNA inside infected hepatocytes with the age of infection. After viral RNA production starts at time a_0, HCV RNA accumulates until the maximal capacity of the cell, $H_{\max}$, is reached at time $a_{\max}$. We assume that a_0 varies between $6\,h$ and $24\,h$. The maximal amount of intracellular viral RNA is generally estimated to be reached on average in less than $a_{\max} \leq 7$ days p.i. .

References

[1] Guedj J, Neumann AU 2010. Understanding hepatitis C viral dynamics with direct-acting antiviral agents due to the interplay between intracellular replication and cellular infection dynamics. J Theor Biol 267: 330–340.

[2] Guo JT, Bichko VV, Seeger C 2001. Effect of alpha interferon on the hepatitis C virus replicon. J Virol 75: 8516–8523.

[3] Guedj J, Dahari H, Rong L, Sansone ND, Nettles RE, Cotler SJ, Layden TJ, Uprichard SL, Perelson AS 2013. Modeling shows that the NS5A inhibitor daclatasvir has two modes of action and yields a shorter estimate of the hepatitis C virus half-life. Proc Natl Acad Sci USA 110: 3991–3996.

[4] Quinkert D, Bartenschlager R, Lohmann V 2005. Quantitative analysis of the hepatitis C virus replication complex. J Virol 79: 13594–13605.

[5] Dahari H, Ribeiro RM, Rice CM, Perelson AS 2007. Mathematical modeling of subgenomic hepatitis C virus replication in Huh-7 cells. J Virol 81: 750–760.
