# Supplementary material for: Inferring Viral Dynamics in Chronically HCV Infected Patients from the Spatial Distribution of Infected Hepatocytes
Source: PLoS Comput Biol. 2014 Nov 13;10(11):e1003934. doi: 10.1371/journal.pcbi.1003934 (PMC4230741; doi:10.1371/journal.pcbi.1003934)
Supplement: Text S3 — Details on simulation and experimental methods. Details on the simulation method to simulate cluster expansion and extended discussion of the experimental method. (PDF) [file pcbi.1003934.s007.pdf]

S3 Details on simulation and experimental methods

S3.1 Simulating cluster expansion

In order to simulate cluster expansion, we use a two-dimensional cellular automaton model consisting of 100×100 hepatocytes distributed in a regular grid. We distinguish between uninfected, infected and infectious hepatocytes. Each simulation starts with a grid of uninfected hepatocytes, and the founding cell of a foci is seeded at time 0 in the middle of the grid.

We assume that infected cells have to pass an eclipse phase of average duration τ before they become infectious. To our knowledge, it has not been thoroughly shown so far if cell-to-cell transmission in HCV infection requires the production and assembling of virions or if transmission can also occur by unpackaged viral RNA only [1]. Recent *in vitro* experiments indicate that HCV cell-to-cell transfer can be blocked by molecules targeting HCV entry receptors such as CD81 and CLDN1 ([2] and Susan Uprichard, *unpublished results*), arguing for a transfer of intact virions during cell-to-cell transmission. Nevertheless, the parameter τ could also be seen as the average duration until viral production starts neglecting viral assembly and packaging. Estimates for both situations are in comparable ranges [3].

At each time step, an infected hepatocyte can become infectious with probability $p_{ii} = 1 - \exp(-1/\tau)$. We assume different average durations of the eclipse phase with $\tau = 6\,h, 12\,h$ or $24\,h$. Once infectious, each cell will start producing and accumulating intracellular viral RNA assuming an average viral RNA production rate of $\alpha = 100\,\text{day}^{-1}$ per infectious cell [4]. As we are examining cluster expansion, we only allow local infection of uninfected neighbors of infected cells. At each time step, an infectious cell infects an uninfected neighbor with transmission probability β_{cc}. We vary the value of the transmission probability β_{cc} between $\beta_{cc} = 10^{-5}$ per RNA^{+} min^{-1} and $\beta_{cc} = 10^{-1}$ per RNA^{+} min^{-1}. Values of $\beta_{cc} \geq 10^{-3}$ per RNA^{+} min^{-1} lead to foci size distributions that are by far larger than the observed foci sizes. Each simulation is followed over a total of $72\,h$ and the simulations that have reached a size of at least 9 cells with radial spread are analyzed. The time step of the simulation is one minute. For each scenario, the expansion of 1000 individual foci is simulated. The simulations are programmed in $\mathbb{C}$++. Analysis is performed using the $\mathbb{R}$ language of statistical computing [5].

S3.2 Precision of scLCM measurements

In order to establish single-cell equivalency of LCM, we extracted RNA and DNA from bulk liver tissue. By quantifying the number of DNA copies of ERV-3 using qPCR, an endogenous retroviral element of fixed copy number in all human cells, we accurately quantified the number of cells in the bulk preparation. We then quantified the amount of 7SL RNA in the same bulk preparation, establishing a standard amount of 7SL per cell. This quantity [compare Fig.2 in [6], red line] was not different from the average amount of 7SL we obtained from scLCM of 1000 individual hepatocytes [green line]. To discern full hepatocytes from cell fragments, two approaches were used. The first was in hepatocyte acquisition: the laser was targeted to the junction between adjacent cell membranes. The second approach was quantitative: by quantifying the amount of 7SL in surrounding PEN membrane, where there was no tissue, we established a relevant negative control. We used the conservative estimate that whole hepatocytes would need to contain greater than five

times the standard deviation above the mean 7SL amounts in the negative controls; therefore, we reasoned that to misclassify a cell fragment as a whole cell would require it to contain more 7SL than 99.9999% of other cell fragments. This approach affords confidence that scLCM is not likely to include cellular fragments in the analysis.

It is possible, however, that non-hepatocyte cellular material was included in the dissections of hepatocytes, as we did not use a negative selective method to prevent this occurrence. It should be stressed, however, that our approach is not different from other in situ approaches in that regard: namely, cell-specific staining is only relatively, so depending on the quality of the antibody block and the visual resolution. Notwithstanding the inherent problems in immunohistochemistry and *in situ* hybridization, per cell measurements are routinely reported as convention, partly because the technique has been in use for several decades.

We would argue that our method has more rigorously established controls to ensure precision than conventional methods. It is very likely that in the future a higher resolution tool will be developed that improves upon our accuracy of single cell measurements.

References

[1] Timpe JM, Stamataki Z, Jennings A, Hu K, Farquhar MJ, Harris HJ, Schwarz A, Desombere I, Roels GL, Balfe P, McKeating JA 2008. Hepatitis C virus cell-cell transmission in hepatoma cells in the presence of neutralizing antibodies. Hepatology 47: 17–24.

[2] Martin DN, Uprichard SL 2013. Identification of transferrin receptor 1 as a hepatitis C virus entry factor. Proc Natl Acad Sci USA 110: 10777–10782.

[3] Keum SJ, Park SM, Park JH, Jung JH, Shin EJ, Jang SK 2012. The specific infectivity of hepatitis C virus changes through its life cycle. Virology 433: 462–470.

[4] Ribeiro RM, Li H, Wang S, Stoddard MB, Learn GH, Korber BT, Bhattacharya T, Guedj J, Parrish EH, Hahn BH, Shaw GM, Perelson AS 2012. Quantifying the diversification of hepatitis C virus (HCV) during primary infection: estimates of the in vivo mutation rate. PLoS Pathog 8: e1002881.

[5] Team RDC 2006. R: A Language and Environment for Statistical Computing. Vienna, Austria: R Foundation for Statistical Computing.

[6] Kandathil AJ, Graw F, Quinn J, Hwang HS, Torbenson M, Perelson AS, Ray SC, Thomas DL, Ribeiro RM, Balagopal A 2013. Use of Laser Capture Microdissection to Map Hepatitis C Virus-Positive Hepatocytes in Human Liver. Gastroenterology 145: 1404–1413.
